# Uridine Prevents Fenofibrate-Induced Fatty Liver

**DOI:** 10.1371/journal.pone.0087179

**Published:** 2014-01-24

**Authors:** Thuc T. Le, Yasuyo Urasaki, Giuseppe Pizzorno

**Affiliations:** 1 Nevada Cancer Institute, Las Vegas, Nevada, United States of America; 2 Desert Research Institute, Las Vegas, Nevada, United States of America; Laurentian University, Canada

## Abstract

Uridine, a pyrimidine nucleoside, can modulate liver lipid metabolism although its specific acting targets have not been identified. Using mice with fenofibrate-induced fatty liver as a model system, the effects of uridine on liver lipid metabolism are examined. At a daily dosage of 400 mg/kg, fenofibrate treatment causes reduction of liver NAD^+^/NADH ratio, induces hyper-acetylation of peroxisomal bifunctional enzyme (ECHD) and acyl-CoA oxidase 1 (ACOX1), and induces excessive accumulation of long chain fatty acids (LCFA) and very long chain fatty acids (VLCFA). Uridine co-administration at a daily dosage of 400 mg/kg raises NAD^+^/NADH ratio, inhibits fenofibrate-induced hyper-acetylation of ECHD, ACOX1, and reduces accumulation of LCFA and VLCFA. Our data indicates a therapeutic potential for uridine co-administration to prevent fenofibrate-induced fatty liver.

## Introduction

Uridine has been widely tested in clinical trials for treatments of neurological disorders, liver dysfunction, and cancer [1]. Uridine is well-known to have positive neurological and systemic effects [1]–[4]. However, lack of understanding of its biological activity hinders effective usage of uridine to modulate human physiology in both healthy and diseased states. Previously, a linkage between pyrimidine biosynthesis pathway and liver lipid metabolism was reported through the use of transgenic uridine phosphorylase 1 (*UPase1*-TG) mice with overexpression of UPase1 and depleted endogenous uridine source [5]. UPase1 is an enzyme that catalyzes the reversible conversion of uridine into uracil and regulates uridine homeostasis [6]. *UPase1*-TG mice exhibited fatty liver phenotype, which could be reversed with dietary uridine supplementation. Uridine was found to modulate liver lipid metabolism although its specific acting targets have not been identified [5].

The liver is an important source of uridine, where circulating plasma uridine is degraded in a single pass and replaced with newly synthesized uridine [7]. Most tissues lack the ability to synthesize uridine and rely on plasma for uridine supply [8]. Thus, the liver serves as an effective regulator of whole-body uridine homeostasis. The concentration of circulating uridine is highly conserved across species of between 2 µM to 4 µM [8], [9]. To maintain circulating uridine homeostasis, the liver has multiple robust means to manage surges in plasma uridine concentration due to dietary intakes. Uridine could be salvaged into pyrimidine nucleotide pool of UTP, CTP, and TTP, or catabolized into β-alanine and acetyl-CoA [1]. Acute surges of uridine or its metabolites in the liver have the ability affect other energy metabolism processes as evidence by the ability of dietary uridine supplementation to modulate liver lipid metabolism [5].

The liver is also a primary site for drug detoxification, which renders it highly susceptible to drug-induced fatty liver [10]. Drug-induced fatty liver is a well-known side effect of many currently FDA-approved drugs [11]–[13]. Most drugs cause fatty liver by inhibiting hepatic fatty acid oxidation [14], [15]. Fatty liver due to chronic drug usage increases the risk for the development of non-alcoholic fatty liver disease such as steatohepatitis and cirrhosis [16], [17]. Current clinical approach to the prevention of fatty liver is dependent on the management of obesity or obesity-associated metabolic diseases, often via pharmaceutical means [18]. However, this approach is problematic when the drugs themselves are contributors to the development of fatty liver condition.

In this study, the ability of uridine to modulate liver lipid metabolism is evaluated in a C57bl/6 mouse model with drug-induced fatty liver. Previously, our lab reported that fenofibrate, when administered at high dosage, induced severe hepatic microvesicular steatosis in mice [19]. Fenofibrate is a peroxisome proliferator-activated receptor-α (PPAR-α) agonist known for its blood lipid-lowering effects [20], [21]. Fenofibrate is widely prescribed for the treatment of dyslipidemia, type 2 diabetes, and the metabolic syndrome [22]. Fenofibrate, via PPAR-α, stimulates the remodeling of hepatic lipid metabolism and promotes fatty acid oxidation [23], [24]. However, inhibitory effects of fenofibrate on fatty acid oxidation have also been reported in rodents at high dosage [25], [26]. Fenofibrate induces hepatocarcinoma and fatty liver in rodents but not in humans [19], [27], [28]. Uridine is co-administered with fenofibrate via dietary supplementation and the effects of uridine on liver lipid metabolism are evaluated in C57bl/6 mice. We aim to evaluate the therapeutic potential of uridine for the prevention of drug-induced fatty liver.

## Results

**Figure 1:**
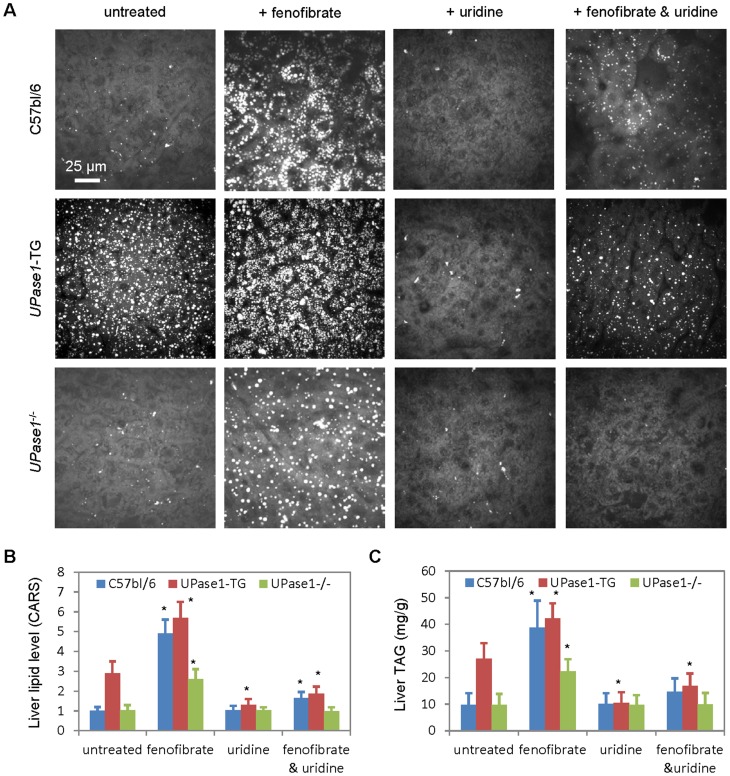
Endogenous and exogenous uridine protects liver against fenofibrate-induced steatosis. (**A**) CARS images of liver tissues of C57bl/6, *UPase1* ^-/-^, and *UPase1*-TG mice in the presence of uridine, fenofibrate, or both uridine and fenofibrate. (**B**) Quantitative analysis of liver lipid level using ImageJ-assisted analysis of CARS images. Liver lipid level is normalized to 1 for control untreated C57bl/6 mice and correspondingly for *UPase1* ^-/-^ and *UPase1*-TG mice or treatment conditions. (**C**) Liver triacylglyceride (TAG) level determined with biochemical assays. Error bars are standard deviations across 9 mice analyzed per animal or treatment group. *P<0.05 versus untreated control.

First, the relationship between endogenous liver uridine concentration and fenofibrate-induced fatty liver was evaluated in C57bl/6 mice and mice with disrupted uridine homeostasis, *UPase1* ^-/-^ and *UPase1*-TG mice of C57bl/6 background. *UPase1* ^-/-^ mice had genetic knock-out of *UPase1* and elevated endogenous liver uridine concentration of 43 µM compared to 6 µM in C57bl/6 mice [6]. In contrast, *UPase1*-TG mice had genetic knock-in of *UPase1* and reduced endogenous liver uridine concentration of 0.5 µM [5]. Using CARS microscopy as a sensitive means to visualize liver lipid [19], [29], [30], both C57bl/6 mice and *UPase1* ^-/-^ mice did not exhibit any visible fatty liver phenotype (**Figure 1A**). *UPase1*-TG mice with endogenous 0.5 µM of liver uridine concentration exhibited mild microvesicular steatosis [5], [19]. Administration of fenofibrate at 400 mg/kg/day induced a 5-fold and a 3-fold increase in liver lipid content in C57bl/6 and *UPase1* ^-/-^ mice, respectively (**Figure 1B**). On the other hand, administration of fenofibrate induced a 2-fold increase in liver lipid content in *UPase1*-TG mice compared to untreated *UPase1*-TG mice, or a 6-fold increase in liver lipid content compared to untreated C57bl/6 or *UPase1* ^-/-^ mice. Thus, depletion of endogenous liver uridine concentration aggravated, whereas elevation of endogenous liver uridine concentration alleviated, fenofibrate-induced fatty liver.

Next, the impact of exogenous uridine supplementation on fatty liver phenotype was evaluated in C57bl/6, *UPase1* ^-/-^, and *UPase1*-TG mice. Consistent with our previous findings, dietary uridine supplementation at 400 mg/kg/day completely suppressed intrinsic fatty liver phenotype of *UPase1*-TG mice (**Figure 1A–C**) [5]. For fenofibrate-treated C57bl/6 mice, uridine supplementation completely suppressed fatty liver phenotype of *UPase1* ^-/-^ mice. Uridine supplementation reduced 70% of liver lipid content of fenofibrate-treated C57bl/6 and *UPase1*-TG mice. Clearly, the protective effect against fatty liver phenotype was exerted by both endogenous and exogenous uridine sources.

**Figure 2:**
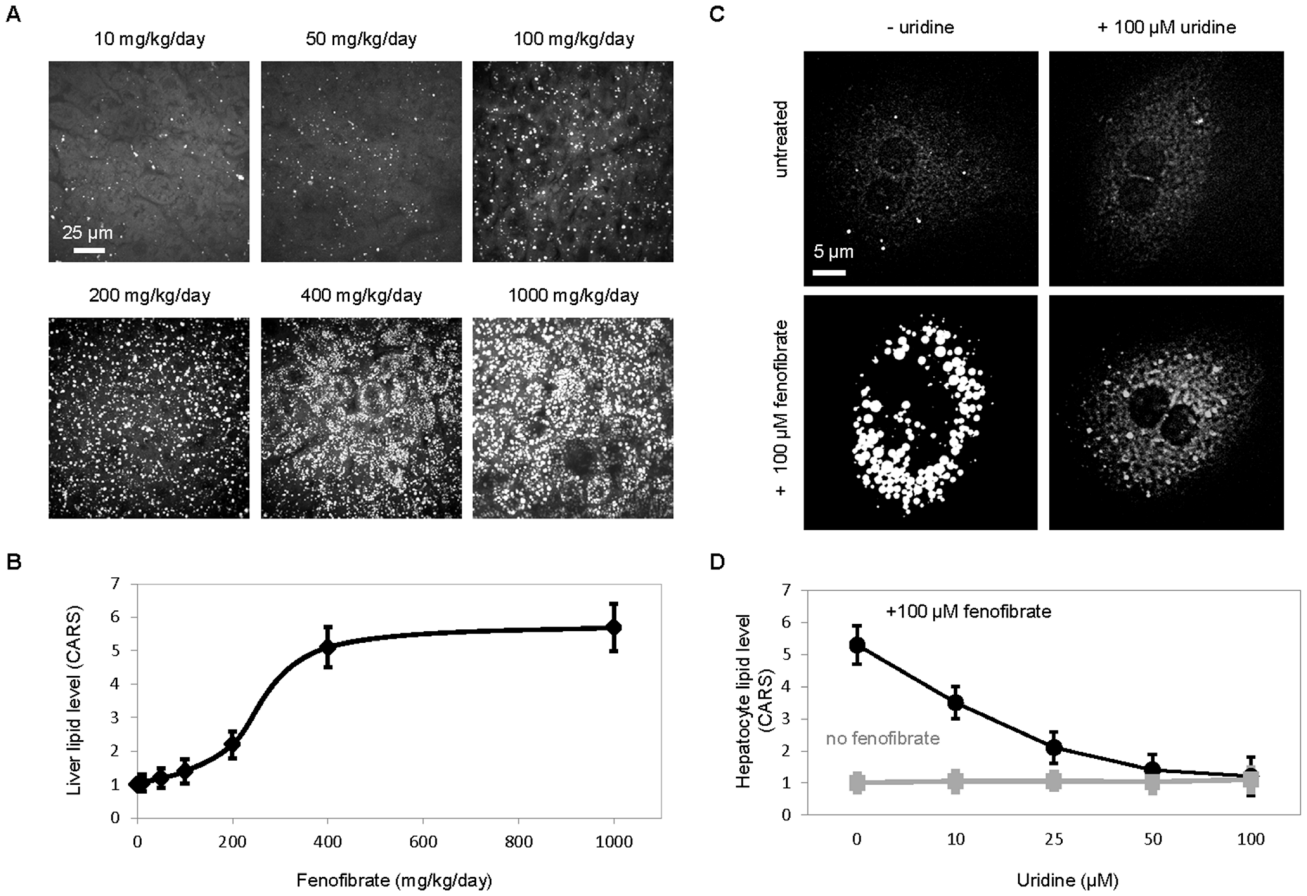
Dose-dependent effects of fenofibrate and uridine. (**A**) CARS images of liver tissues of C57bl/6 mice treated with different fenofibrate dosages. (**B**) Quantitative analysis of liver lipid level using ImageJ-assisted analysis of CARS images. Error bars are standard deviations across 3 mice analyzed per fenofibrate dosage. (**C**) CARS images of C57bl/6 primary hepatocytes treated with variable concentrations of uridine alone (grey) or with 100 µM fenofibrate together with variable concentrations of uridine (black). (**D**) Quantitative analysis of hepatocyte lipid level using ImageJ-assisted analysis of CARS images. Error bars are standard deviations across 60 hepatocytes analyzed per uridine concentration.

The effective dosages of fenofibrate to induce and uridine to prevent lipid accumulation were evaluated in mice and primary hepatocytes, respectively. C57bl/6 mice were fed with different dosages of fenofibrate and the lipid content of collected liver tissues were examined with CARS microscopy (**Figure 2A**). The half-maximal effective dosage of fenofibrate to induce fatty liver phenotype was determined to be approximately 250 mg/kg/day (**Figure 2B**). On the other hand, the effective concentration of uridine to prevent fenofibrate-induced lipid accumulation was evaluated in freshly collected primary hepatocyte cultures (**Figure 2C**). The half-maximal effective concentration of uridine to suppress fenofibrate-induced lipid accumulation in primary hepatocytes was determined to be approximately 20 µM (**Figure 2D**).

**Figure 3:**
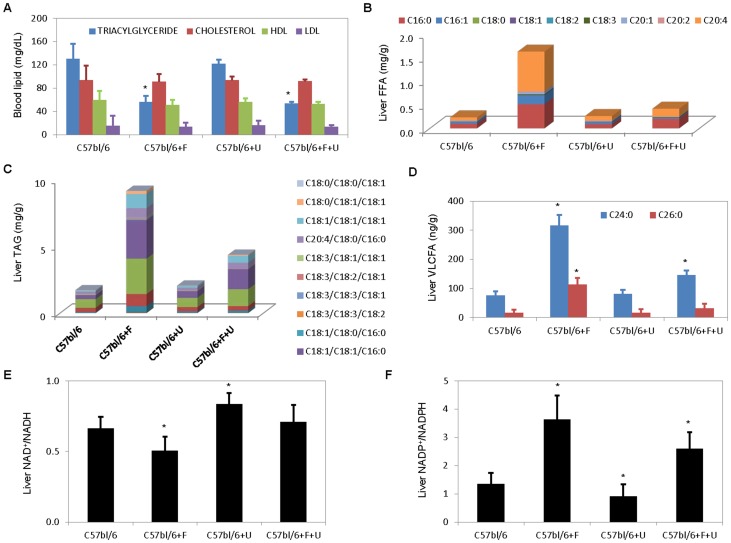
Evaluation of blood and liver lipids and liver NAD^+^/NADH and NADP^+^/NADPH ratios. (**A**) Blood level of triacylglyceride (TAG), cholesterol, high-density lipoprotein (HDL), and low-density lipoprotein (LDL) in control and treated C57bl/6 mice. (**B–D**) LC-MS analysis of liver (**B**) free fatty acids (FFA), (**C**) TAG, and (**D**) very long chain fatty acids (VLCFA). All data present in **A–D** are average of 3 mice analyzed per treatment group. (**E–F**) Liver (**E**) NAD^+^/NADH and (**F**) NADP^+^/NADPH ratios measured with biochemical assays. Error bars are standard deviations across 9 mice evaluated per treatment group. *P<0.05 versus untreated control.

To complement CARS microscopy studies, characterizations of blood and liver tissues using established biochemical assays were also performed. Fenofibrate treatment at 400 mg/kg/day was effective at lowering blood TAG level by more than 50% in C57bl/6 mice (**Figure 3A**). Blood cholesterol, HDL, and LDL levels were statistically unchanged with fenofibrate treatment. Fenofibrate is known to reduce total blood cholesterol and LDL levels and raise HDL level in both human and rodents with dyslipidemia [22], [31]. However, C57bl/6 mice used in our experiments were 10–12 weeks old with normal blood lipid levels. It is possible that the blood-lipid lowering effects of fenofibrate were most prominent with TAG levels and less so with cholesterol, LDL, and HDL levels in healthy young mice. Uridine supplementation had no effect on blood TAG and cholesterol levels in C57bl/6 mice. Neither did uridine supplementation have any effect on TAG and cholesterol levels of mice treated with fenofibrate.

Next, LC-MS was employed to measure FFA and TAG species from liver total lipid extracts (**Figure 3B, C**). Overall, LC-MS measurements concurred with CARS microscopy in term of liver lipid phenotype induced by fenofibrate treatment and the protective effect of uridine supplementation against liver lipid accumulation. Most interestingly, fenofibrate treatment was associated with the accumulation of long chain fatty acid fatty acid (LCFA) C20:4 and very long chain fatty acids (VLCFA) C24:0 and C26:0 (**Figure 3B, D**). Uridine supplementation reduced liver LCFA and VLCFA accumulation in fenofibrate treated mice. Accumulation of LCFA and VLCFA is a clinical indication of peroxisomal β-oxidation impairment [32]–[34]. Because fenofibrate exerts its effect on peroxisomal proliferation, it is possible that peroxisomal biogenesis or function might play a role in fenofibrate-induced fatty liver.

Furthermore, significant changes to the cellular reduction-oxidation potential associated with fenofibrate treatment were detected with biochemical assays (**Figure 3E, F**). Liver tissues of mice treated with fenofibrate exhibited a 25% reduction in the NAD^+^/NADH ratio and a 270% increase in the NADP^+^/NADPH ratio compared to untreated mice. Uridine supplementation alone caused a 26% increase of NAD^+^/NADH ratio and a 33% reduction of NADP^+^/NADPH ratio in the liver tissues of C57bl/6 mice. In mice treated with fenofibrate, uridine supplementation completely restored the NAD^+^/NADH ratio back to the level observed in untreated C57bl/6 mice. On the other hand, uridine supplementation reduced fenofibrate-induced increase of NADP^+^/NADPH ratio by approximately 30%. Clearly, fenofibrate treatment induced imbalance to hepatic cellular reduction-oxidation potential of the liver tissues by altering NAD^+^/NADH and NADP^+^/NADPH ratios. Uridine supplementation mitigated such effects and partially restored hepatic cellular reduction-oxidation potential.

**Figure 4:**
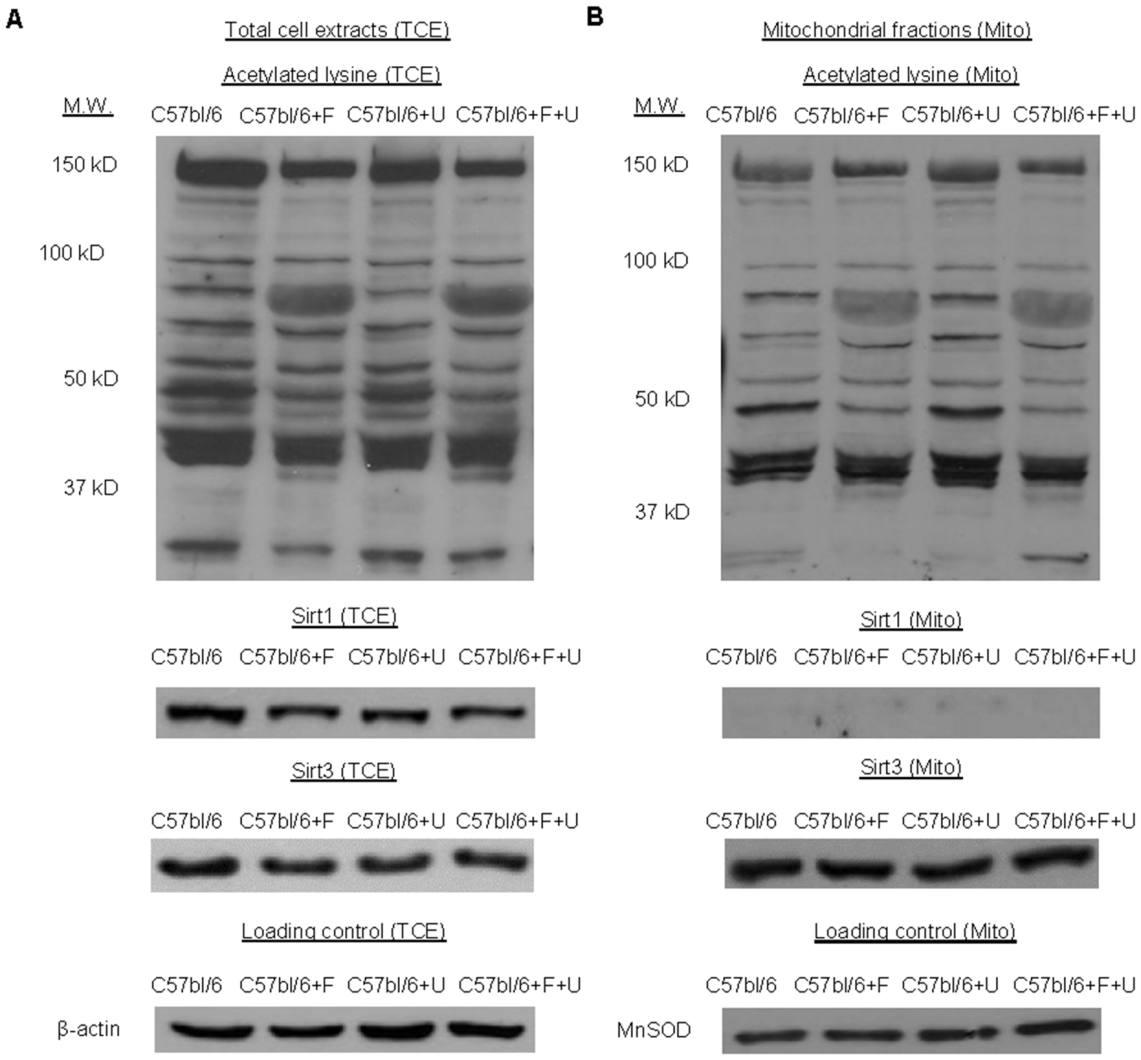
1D Western blots of acetylated proteins, Sirt1, and Sirt3 of liver (A) total cell extracts (TCE) and (B) mitochondrial fractions (Mito). β-actin and MnSOD were used as loading controls for TCE and Mito, respectively. Data are representative of 1D WB analyses of 3 mice per treatment group.

Previously, we showed that uridine supplementation has the ability to affect lysine acetylation profiles of metabolic and redox enzymes [5]. Here, we examined the effects of fenofibrate and uridine treatment on liver protein acetylation profiles. Using 1D Western blots of total liver cell extracts with antibodies against acetylated lysine, we found that fenofibrate induced over-acetylation of a protein band with molecular weight of approximately 80 kD (**Figure 4A**). 1D Western blots of liver mitochondrial fractions revealed that this protein band was associated with the mitochondrial fractions (**Figure 4B**). Uridine supplementation did not appear to affect the acetylation profile of this protein band on 1D Western blots.

**Figure 5:**
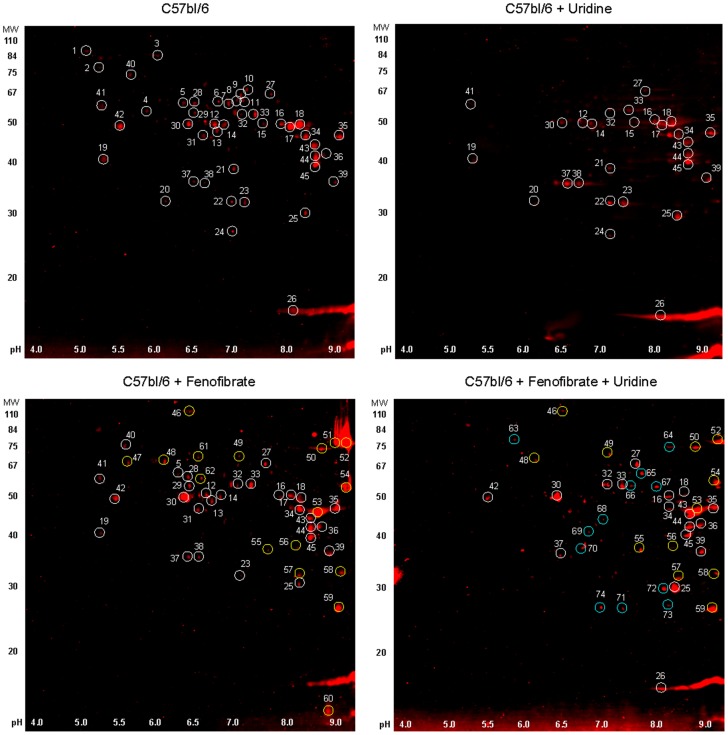
2D Western blots of acetylated proteins in liver total cell extracts. White circles mark the acetylated protein spots presence in untreated samples. Yellow circles mark the acetylated protein spots presence in fenofibrate treated samples but not in untreated WT samples. Cyan circles mark the acetylated protein spots presence in uridine and fenofibrate treated samples but not in untreated samples or samples treated with fenofibrate alone. 2D Western blots were performed by Applied Biomics.

Using a proteomics approach with 2D Western blots to analyzed acetylated proteins followed by MALDI-TOF-MS identification of interested protein spots, two acetylated peroxisomal proteins, ECHD and ACOX1, were found among other acetylated proteins with molecular weights of 80 kD (**Figure 5** **,** **Table 1** **,** **Table 2** **& Figure S1**). In liver tissues of mice treated with fenofibrate, over-acetylation of ECHD and ACOX1 proteins were observed. Co-administration of uridine with fenofibrate significantly reduced the levels of acetylation of liver ECHD and ACOX1 proteins. ECHD and ACOX1 proteins were present in the liver mitochondrial fraction because liver peroxisomes normally get separated together with liver mitochondria in most of mitochondria isolation protocols. Indeed, a previous proteomic study of mitochondrial acetylome identified a large number of peroxisomal proteins including ECHD and ACOX1 proteins [35]. The presence of ECHD and ACOX1 proteins at multiple gel spots with different molecular weights and/or pH values was most likely due to post-translational modifications or degradation. The inability to observe the effect of uridine on lysine acetylation of proteins with 80 kD molecular weight on 1D Western blots was likely due to lack of resolution for protein separation.

**Table 1:** Liver acetylated proteins identified with MALDI-TOF-MS.

Spot #	Protein Name	C57bl/6	C57bl/6+F	C57bl/6+U	C57bl/6+F+U	Biological Process
1	Endoplasmin	√	-	-	-	ER Molecular Chaperone
2	78 kDa glucose-regulated protein	√	-	-	-	Assembly of ER Protein Complexes
3	Cytosolic 10-formyltetrahydrofolate dehydrogenase	√	-	-	-	ER Overload Response
4	S-adenosylmethionine synthase isoform type-1	√	-	-	-	One Carbon Metabolism
5	Aldehyde dehydrogenase, mitochondrial	√	√	-	-	Oxidation-Reduction
6	Glutamate dehydrogenase 1, mitochondrial	√	-	-	-	Amino Acid Metabolism
7	Glutamate dehydrogenase 1, mitochondrial	√	-	-	-	Amino Acid Metabolism
8	Dihydropyrimidinase	√	-	-	-	Pyrimidine Metabolism
9	Catalase	√	-	-	-	Antioxidation
10	Delta-1-pyrroline-5-carboxylate dehydrogenase, mitochondrial	√	-	-	-	Urea/TCA Cycle
11	Methylmalonate-semialdehyde dehydrogenase, mitochondrial	√	-	-	-	Pyrimidine Metabolism
12	SEC14-like protein 2	√	√	√	-	Cholesterol Biosynthesis
13	Fumarylacetoacetase	√	√	-	-	Phe/Tyr Catabolism
14	Isocitrate dehydrogenase [NADP] cytoplasmic	√	√	√	-	TCA Cycle
15	Argininosuccinate synthase	√	-	√	-	Amino Acid Metabolism
16	Betaine–homocysteine S-methyltransferase 1	√	√	√	√	Amino Acid Metabolism
17	Argininosuccinate synthase	√	√	√	-	Amino Acid Metabolism
18	Argininosuccinate synthase	√	√	√	√	Amino Acid Metabolism
19	Regucalcin	√	√	√	-	Calcium Homeostasis
20	Indolethylamine N-methyltransferase	√	-	√	-	Detoxification
21	Glycine N-methyltransferase	√	-	√	-	One Carbon Metabolism
22	Carbonic anhydrase 3	√	-	√	-	Acid-Base Balance
23	Carbonic anhydrase 3	√	√	√	-	Acid-Base Balance
24	Superoxide dismutase [Mn], mitochondrial	√	-	√	-	Antioxidation
25	Glutathione S-transferase	√	√	√	√	Antioxidation
26	Histone H2B type 1-P	√	-	√	√	Nucleosome Assembly
27	Catalase	√	√	√	√	Antioxidation
28	Aldehyde dehydrogenase X, mitochondrial	√	√	-	-	Oxidation-Reduction
29	Alpha-enolase	√	√	-	-	Glycolysis
30	Acyl-coenzyme A thioesterase 1	√	√	√	√	Lipid Metabolism
31	Arginase-1	√	√	-	-	Urea Cycle
32	Hydroxymethylglutaryl-CoA synthase, mitochondrial	√	√	√	√	Steroid Metabolism
33	Hydroxymethylglutaryl-CoA synthase, mitochondrial	√	√	√	√	Steroid Metabolism
34	Alcohol dehydrogenase 1	√	√	√	√	Oxidation-Reduction
35	3-ketoacyl-CoA thiolase B, peroxisomal	√	√	√	√	Lipid Metabolism
36	Malate dehydrogenase, mitochondrial	√	√	-	√	TCA cycle
37	Cytochrome c1, heme protein, mitochondrial	√	√	√	√	Electron Transport
38	L-xylulose reductase	√	√	√	-	Glucose Metabolism

**Table 2:** Liver acetylated proteins identified with MALDI-TOF-MS (continued).

Spot #	Protein Name	C57bl/6	C57bl/6+F	C57bl/6+U	C57bl/6+F+U	Biological Process
39	D-beta-hydroxybutyrate dehydrogenase, mitochondrial	√	√	√	√	Ketone Bodies Metabolism
40	Heat shock cognate 71 kDa protein	√	√	-	-	Stress Response
41	ATP synthase subunit beta, mitochondrial	√	√	√	-	ATP Biosynthesis
42	Actin, cytoplasmic 2	√	√	-	√	Cytoskeleton
43	Fructose-bisphosphate aldolase B	√	√	√	√	Glycolysis
44	Glyceraldehyde-3-phosphate dehydrogenase	√	√	√	√	Glycolysis
45	Uricase	√	√	√	√	Purine Metabolism
46	Carbamoyl-phosphate synthase [ammonia], mitochondrial	-	√	-	√	Pyrimidine Metabolism
47	60 kDa heat shock protein, mitochondrial	-	√	-	-	Stress Response
48	Epoxide hydrolase 2	-	√	-	√	Lipid Metabolism/Detoxification
49	NADP-dependent malic enzyme	-	√	-	√	Malate Metabolism
50	Peroxisomal acyl-coenzyme A oxidase 1	-	√	-	√	Lipid Metabolism
51	Peroxisomal bifunctional enzyme	-	√	-	-	Lipid Metabolism
52	Peroxisomal bifunctional enzyme	-	√	-	√	Lipid Metabolism
53	3-ketoacyl-CoA thiolase B, peroxisomal	-	√	-	√	Lipid Metabolism
54	Elongation factor 1-alpha 1	-	√	-	√	Protein Biosynthesis
55	Glycine N-acyltransferase-like protein	-	√	-	√	Acyltransferase
56	Hydroxyacyl-coenzyme A dehydrogenase, mitochondrial	-	√	-	√	Lipid Metabolism
57	Electron transfer flavoprotein subunit beta	-	√	-	√	Electron Transport
58	Protein NipSnap homolog 1	-	√	-	√	Unknown
59	Peroxisomal acyl-coenzyme A oxidase 1	-	√	-	√	Lipid Metabolism
60	Fatty acid-binding protein, liver	-	√	-	-	Lipid Metabolism/FA Transport
61	Phosphoglucomutase-1	-	-	-	√	Glucose Metabolism
62	4-trimethylaminobutyraldehyde dehydrogenase	-	-	-	√	Carnitine Biosynthesis
63	Protein disulfide-isomerase A4	-	-	-	√	Protein Folding
64	Peroxisomal acyl-coenzyme A oxidase 1	-	-	-	√	Lipid Metabolism
65	Retinal dehydrogenase 1	-	-	-	√	Retinol Metabolism
66	Hydroxymethylglutaryl-CoA synthase, mitochondrial	-	-	-	√	Steroid Metabolism
67	Hydroxymethylglutaryl-CoA synthase, mitochondrial	-	-	-	√	Steroid Metabolism
68	Alcohol dehydrogenase [NADP+]	-	-	-	√	Oxidation-Reduction
69	Glycerol-3-phosphate dehydrogenase [NAD+]	-	-	-	√	Glycolysis
70	S-formylglutathione hydrolase	-	-	-	√	Formaldehyde Catabolism
71	Superoxide dismutase [Mn], mitochondrial	-	-	-	√	Oxidation-Reduction
72	Glutathione S-transferase	-	-	-	√	Oxidation-Reduction
73	Glutathione S-transferase	-	-	-	√	Oxidation-Reduction
74	Peptidyl-tRNA hydrolase 2, mitochondrial	-	-	-	√	Apoptosis

MALDI-TOF-MS identification of 74 acetylated protein spots revealed that most acetylated proteins participated in cellular metabolism, oxidation-reduction, electron transport, and detoxification (**Table 1** **,** **Table 2** **& Tables S1–S3**). Most notable was the presence of acetylated FABP-1, a small cytoplasmic protein that binds to long chain fatty acids and participates in fatty acid uptake, transport, and metabolism [36]. FABP-1 was heavily acetylated in the liver tissues of mice treated with fenofibrate. Uridine co-administration with fenofibrate prevented over-acetylation of liver FABP-1 protein. The acetylation of ECHD, ACOX1, FABP-1, and some other selected proteins were subjected to further analysis for acetylation sites. It was determined that indeed lysine acetylated proteins identified with 2D WB had multiple acetylation sites identified with MALDI-TOF-MS-MS (**Tables S4–S6**).

**Figure 6:**
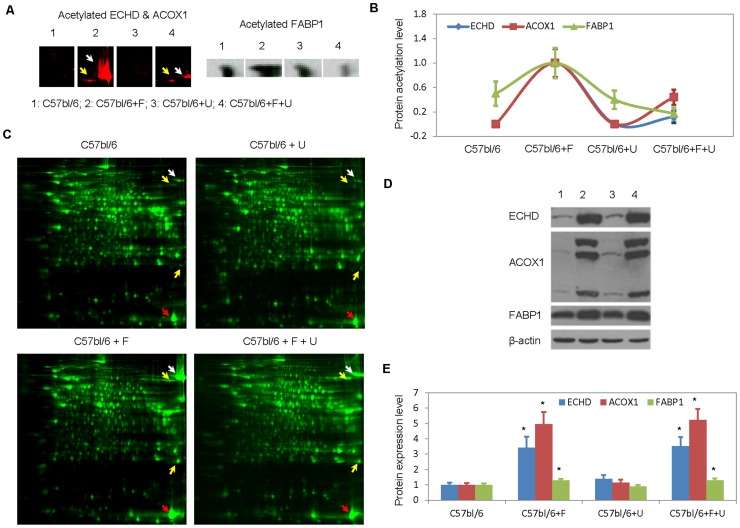
Protein expression and acetylation levels of ECHD, ACOX1, and FABP1. (**A**) Lysine acetylation of ECHD, ACOX1, and FABP1 detected with 2D Western blots. White arrows: ECHD; yellow arrows: ACOX1. (**B**) Quantitative analysis of protein acetylation levels on 2D Western blots. Data are normalized to 1 for liver samples with fenofibrate treatment and respectively for control and other treatment groups. Error bars are standard deviation of duplicate measurements. Only the ACOX1 band at 80 kD and pH 9 was used for acetylation level analysis. (**C**) 2D protein gels of liver total cell extracts. White arrows: ECHD; yellow arrows: ACOX 1; red arrows: FABP1. (**D**) 1D Western blot using antibodies direct against ECHD, ACOX1, or FABP1. β-actin serves as the loading control. (**E**) Quantitative analysis of protein expression levels on 1D WB gels. Error bars are standard deviation of triplicate measurements. Data were normalized to 1 for untreated control samples and respectively for treated samples. *P<0.05 versus untreated control.

Uridine supplementation affected the acetylation of ECHD, ACOX1, and FABP-1 but not their expression levels. In the liver tissues of mice treated with fenofibrate, increases acetylation of ECHD, ACOX1, and FABP-1 were observed (**Figure 6A, B**). Uridine co-administration significantly reduced fenofibrate-induced acetylation of liver ECHD, ACOX1, and FABP-1. Fenofibrate treatment also increased the protein expression levels of ECHD, ACOX1, and FABP-1 (**Figure 6C–E**). However, uridine administration by itself or together with fenofibrate had no effect on the expression levels of ECHD, ACOX1, and FABP-1. Fenofibrate is an agonist of PPARα, a transcription factor that regulates the expression of genes encoding for ECHD, ACOX1, and FABP-1 [37]. Our data suggests that uridine did not interfere with the interaction between fenofibrate and PPARα.

**Figure 7:**
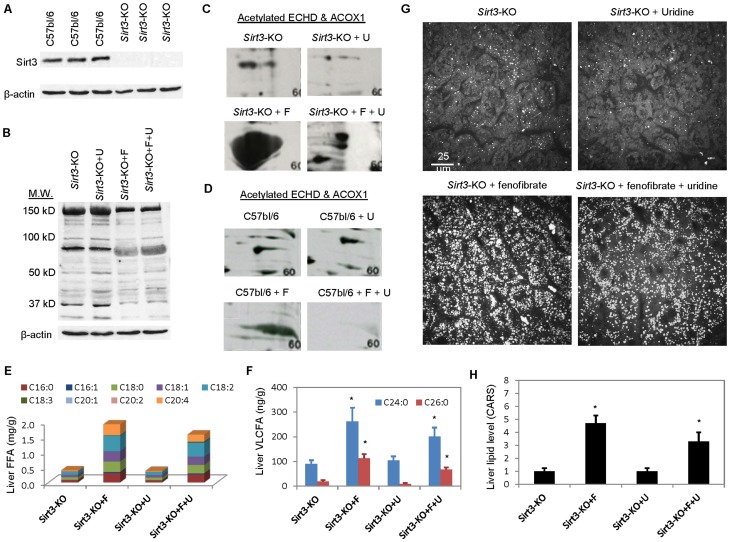
Uridine is ineffective in preventing fenofibrate-induced fatty liver in *Sirt3*-KO mice. (**A**) Western blot analysis of Sirt3 protein expression in C57bl/6 and *Sirt3*-KO mice. (**B**) 1D Western blot analysis of liver lysine acetylation profiles as a function of uridine and/or fenofibrate treatment in *Sirt3*-KO mice. 2D Western blot analysis of lysine acetylation profiles of ECHD and ACOX1 as a function of uridine and/or fenofibrate treatment in *Sirt3*-KO mice (**C**) and in C57bl/6 mice (**D**). 2D Western blots were performed by Kendrick Laboratories. LC-MS analyses of liver FFA (**E**) and VLCFA (**F**). (**G**) CARS images of *Sirt3*-KO liver tissues as a function of uridine and/or fenofibrate treatment. (**H**) Quantitative analysis of liver lipid level using ImageJ-assisted analysis of CARS images. Liver lipid level is normalized to 1 for control untreated *Sirt3*-KO mice and correspondingly for uridine and/or fenofibrate treatment. *P<0.05 versus untreated control.

To further explore the relationship between uridine co-administration and protein acetylation, a *Sirt3*-KO mouse model was employed. *Sirt3*-KO mice had targeted deletion of exon 2–3 of the mouse sirtuin homolog 3, *Sirt3*, gene; therefore, abolished *Sirt3* gene function [38]. Sirt3 is a NAD^+^-dependent protein deacetylase that regulates global mitochondrial protein acetylation [38]. Sirt3 regulates mitochondrial fatty acid oxidation by controlling acetylation state of mitochondrial proteins [39]. Sirt3 deficiency in Sirt3-KO mice is associated with accelerated development of metabolic syndrome [40]. Consistently, expression of Sirt3 protein was lacking in *Sirt3*-KO mice compared to C57bl/6 mice when analyzed with Western blots (**Figure 7A**). Fenofibrate treatment also induced hyper-acetylation to proteins with molecular weights of approximately 80 kD, which was detectable with 1-D Western blots (**Fig. 7B**). 2-D Western blots revealed that fenofibrate induced hyper-acetylation of proteins that have isoelectric points and molecular weights of ECHD and ACOX1 (**Figure 7C** **& Figure S2**). Uridine co-administration with fenofibrate significantly reduced acetylation of these proteins. When compared similar fenofibrate and uridine co-treatments between mice strains, significant more acetylation of ECHD and ACOX1 remained in *Sirt3*-KO mice compared to C57bl/6 mice (**Figure 7** **C, D, Figure S1 & Figure S2**). This observation indicated that while Sirt3 might play a role in mediating uridine-induced protein deacetylation, participation of deacetylases other than Sirt3 were likely. Indeed, mammals possess 7 sirtuins (Sirt1–7), where 3 sirtuins are associated with the mitochondrial fractions (Sirt3, -4, and -5) [41], [42].

Uridine co-administration was less effective in *Sirt3*-KO mice in preventing fenofibrate-induced fatty liver. Analysis of FFA species with LC-MS revealed that fenofibrate treatment induced accumulation of liver LCFA and VLCFA in *Sirt3*-KO (**Figure 7E, F**). Co-administration of uridine with fenofibrate partially prevented accumulation of liver LCFA and VLCFA; however, significant liver LCFA and VLCFA remained in *Sirt3*-KO mice. CARS imaging of liver lipid level and subsequently quantitative analysis concurred with the observation made with LC-MS measurements (**Figure 7G, H**). Due to the small molecular weight of FABP1 of approximately 14 kD, which migrated at the edge of the 2D gels, analysis of its acetylation levels in *Sirt3*-KO mice as a function of fenofibrate and/or uridine treatment was inconclusive.

**Figure 8:**
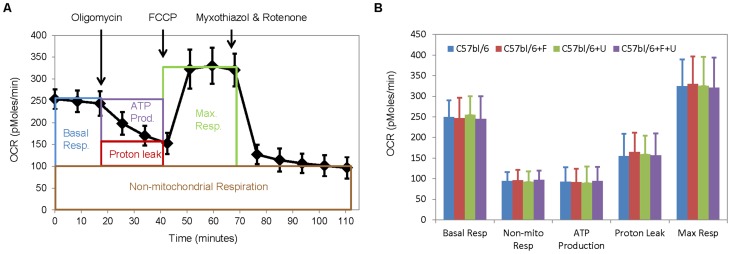
Bioenergetics of primary hepatocytes. (**A**) An example of the mitochondrial function profiles of primary hepatocytes evaluated with a stress test kit. (**B**) Oxygen consumption rates (OCR) as a function of control and experimental C57bl/6 primary hepatocyte cultures. Error bars are standard deviations of 12 repeated measurements.

Uridine has been reported to have no impact on mitochondrial function in healthy biological systems [43]. In cases of drug-induced mitochondrial dysfunction, uridine was found to improve mitochondrial function via replenishment of pyrimidine nucleotide pools [43] or via mechanisms beyond the pyrimidine deficit [44]. To evaluate the effects of uridine on mitochondrial respiration, an Extracellular Flux Analyzer was employed to measure oxygen consumption rates of isolated primary hepatocyte cultures. Following previously described protocols, 5 key parameters of cellular bioenergetics were measured, including basal respiration, non-mitochondrial respiration, ATP production, proton leak, and maximal respiration (**Figure 8A**) [45]. Fenofibrate administration by itself or in combination with uridine had no effect on the bioenergetics of primary hepatocytes (**Figure 8B**). Neither did uridine administration by itself have any impact on the bioenergetics of primary hepatocytes. Alternatively, mitochondrial fatty acid β-oxidation of tritiated palmitic acid was also measured in primary hepatocyte cell cultures (**Figure S3**). Neither fenofibrate nor uridine was found to affect mitochondrial fatty acid β-oxidation. Thus, neither fenofibrate nor uridine, individually or together, modulated liver lipid accumulation by acting directly on mitochondrial function.

## Discussion

In this study, the ability of uridine to prevent fenofibrate-induced fatty liver in mice was examined. Using CARS microscopy as a sensitive means to monitor liver lipid, fenofibrate was found to induce fatty liver with an EC_50_ value of 200 mg/kg daily dosage. Interestingly, dietary uridine supplementation at 400 mg/kg daily dosage suppressed fenofibrate-induced fatty liver phenotype. The EC_50_ value for uridine to suppress fenofibrate-induced lipid accumulation was approximately 20 µM in cultured primary hepatocytes. Elevated uridine concentration due to both endogenous and exogenous sources exerted protective effects against fenofibrate-induced fatty liver phenotype.

We found that uridine co-administration with fenofibrate was associated with reduced acetylation of ECHD, ACOX1, and FABP-1. Uridine co-administration did not interfere with the ability of fenofibrate to lower blood TAG level. Neither did uridine co-administration interfere with fenofibrate-induced increased expression of ECHD, ACOX1, and FABP-1. Generally, uridine did not have any effect on the expression level of lipid metabolism genes as reported previously [5]. Following fenofibrate treatment, ECHD, ACOX1, and FABP-1 became hyper-acetylated. Uridine co-administration with fenofibrate was associated with reduced acetylation of ECHD, ACOX1, and FABP-1. Most interestingly, uridine co-administration reduced fenofibrate-induced accumulation of liver LCFA and VLCFA. Peroxisomes are the organelles that carry out β-oxidation of LCFA and VLCFA [34]. ECHD and ACOX1 are critical enzymes for peroxisomal fatty acids β-oxidation. FABP-1 is a cytoplasmic protein that shuttles LCFA and VLCFA to and from cellular organelles [36]. It is plausible that hyper-acetylation of ECHD, ACOX1, and FABP-1 inhibits their functions and causes impairment of peroxisomal fatty acid β-oxidation, leading to the accumulation of LCFA and VLCFA [46].

Both uridine and fenofibrate have the capacity to affect liver protein lysine acetylation. Fenofibrate is known to stimulate fatty acid catabolism by activating PPARα [34]. A direct product of fatty acid catabolism is acetyl-CoA, which serves as a donor for protein lysine acetylation [47]. Indeed, we found that fenofibrate treatment at 400 mg/kg daily dosage increased liver acetyl-CoA concentration by nearly two folds compared to untreated control, 71 nmol/g versus 40 nmol/g (p<0.01), respectively (**Figure S4**). Such dramatic increase in liver acetyl-CoA concentration following fenofibrate treatment could lead to hyper-acetylation of ECHD, ACOX1, and FABP-1. In fact, non-enzymatic lysine acetylation of proteins is a well-documented phenomenon [48]. Uridine co-administration did not interfere with fenofibrate-induced increases in liver acetyl-CoA concentration (**Figure S4**). However, uridine administration was associated with an increase in NAD^+^/NADH ratio, which activates NAD^+^-dependent protein deacetylases leading to protein deacetylation [5], [41]. Consistently, our proteomics data revealed that administration of uridine and fenofibrate, individually or together, affected liver protein acetylation profiles. Most prominently, uridine co-administration with fenofibrate was associated with reduced acetylation of ECHD, ACOX1, and FABP-1.

The utilization of a *Sirt3*-KO mouse model shows that uridine-induced protein deacetylation is mediated in part by Sirt3. Genetic deletion of *Sirt3* gene partially inhibits the ability of uridine to prevent fenofibrate-induced hyper-acetylation of peroxisomal proteins ECHD and ACOX1 and accumulation of LCFA and VLCFA. *In vitro* experiments reveal that uridine by itself has no effect on Sirt3 or Sirt1 enzymatic activity (data not shown). However, deacetylation activity of Sirt3 and Sirt1 are elevated *in* total liver extracts from C57bl/6 mice treated with uridine compared to total liver extracts from C57bl/6 mice not treated with uridine [5]. Our data suggest that the effects of uridine on protein deacetylases are indirect and likely mediated by liver levels of NAD^+^.

Sirt3 is commonly considered as a mitochondrial protein; however, its presence in the nucleus and cytoplasm has also been reported [49]–[53]. Analysis of mitochondrial acetylome by Fritz *et al*. identified a number of peroxisomal proteins including ECHD and ACOX1 [35]. Acetylation states of these peroxisomal proteins are affected by alcohol treatment as well as the expression of Sirt3 [35]. Many peroxisomal proteins in liver tissues of humans and mice have been found to be acetylated and are regulated by metabolic states [35], [54], [55]. Preliminary immune-staining data from our lab show co-localization of Sirt3 proteins to peroxisomes in HepG2 cells (**Figure S5**). While mitochondrial proteins have been considered as the primary acting targets of Sirt3, the presence of Sirt 3 in peroxisomes and its activity on peroxisomal proteins should not be excluded. Further studies are required to describe peroxisomal acetylome, metabolic regulation of peroxisomal protein acetylation, and the associated peroxisomal NAD^+^-dependent protein deacetylases.

Uridine has long been reported to reverse mitochondrial dysfunction induced by different type of drugs [43], [44]. However, we found that neither fenofibrate nor uridine had any impact on mitochondrial respiration of primary hepatocytes. Instead, the effects of uridine and fenofibrate were observed on lysine acetylation of cytoplasmic and peroxisomal proteins. It is possible that uridine exert multi-targeted effects on multiple organelles including mitochondria and peroxisomes. The targeted effects of uridine might vary as a function of drugs. While further studies are needed to delineate biological activities of uridine, we have identified in this study specific protein targets that uridine can have an impact on their post-translational modification. Protein lysine acetylation is an important process in the regulation of liver energy metabolism [56]. Hundreds to thousands of metabolic proteins with acetylated lysine residues have been identified in liver tissues of mice and humans [54], [55]. However, the role of lysine acetylation in regulating the function of most metabolic proteins has not been elucidated [47], [57]. Future in-depth studies on how lysine acetylation affects the function of ECHD, ACOX1, and FABP-1 could shed light on the etiology of drug-induced fatty liver disease and the potential therapeutic usage of uridine to modulate liver lipid metabolism and prevent drug-induced fatty liver.

## Materials and Methods

### Animals

All animal studies were performed in conformity with the Public Health Service Policy on Humane Care and Use of Laboratory Animals and with the approval of the Animal Care and Use Committees at Nevada Cancer Institute, Desert Research Institute, and Touro University Nevada. All mice used were male at 10–12 weeks of age. C57bl/6 mice and *Sirt3*-KO mice (strain name: 129-*Sirt3^tm1.1Fwa^*/J, stock number: 012755) were purchased from Jackson Lab (Bar Harbor, Maine). *Sirt3*-KO mice were generated by Frederick Alt's lab and described previously [38]. *UPase1* ^-/-^ and *UPase1*-TG mice were generated by our labs and described previously [5], [6]. Control mice were fed with PicoLab Mouse Diet ground pellets (Cat. No. 5058, LabDiet, Brentwood, MO) that provide 4.6 kcal/g and consist of 22% protein and 9% fat. The lipid composition includes cholesterol (200 ppm), linoleic acid (2.32%), linolenic acid (0.21%), arachidonic acid (0.02%), and omega-3 fatty acid (0.32%). The total saturated and monounsaturated fatty acids are 2.72% and 2.88%, respectively. When administered alone or in combination, uridine and fenofibrate were thoroughly mixed with ground pellets with approximate daily dosage of 400 mg/kg (or as specified). Mice were placed on control or supplemented diets for 5 days prior to terminal liver and blood samples collection. Mice were not fasted prior to terminal procedures. All samples were collected at approximately the same time in early mornings. A range of daily dosages of fenofibrate and uridine were evaluated in mice. A daily dosage of 400 mg/kg of fenofibrate was chosen to induce severe hepatic steatosis. A daily dosage of 400 mg/kg of uridine was determined to be sufficient to suppress fenofibrate-induced fatty liver.

### Primary hepatocyte cultures

Mouse hepatocytes were isolated using a two-step collagenase perfusion technique described previously [5], [19], [58]. Hepatocytes were either treated with variable uridine concentrations or treated with 100 µM fenofibrate together with variable uridine concentrations for 24 hours at 37°C and 5% CO_2_.

### CARS imaging of liver tissues

A home-built coherent anti-Stokes Raman scattering (CARS) microscope was used to image lipid using CH_2_ vibrational frequency at 2851 cm^−1^ as described previously [59]. Approximately 20 or 31 frames were taken along the vertical axis at 1-micron increment for volumetric evaluation of lipid content of primary hepatocytes and liver tissues, respectively. Lipid level was the square root of resonant CARS signal intensity, which is the difference between total CARS signal intensity and CARS signal intensity arising from cellular membrane and non-resonant signal [19]. Lipid level was normalized to 1 for control untreated primary hepatocytes or mice and respectively for other mice strains or treatment conditions. Quantitative analysis of lipid level was performed using the NIH ImageJ software. Liver was perfused with phosphate buffered saline prior to collection. Liver tissues were sliced into 200-micron thick sections, transferred into glass-bottom chambered slides, overlaid with 200 microliters of 1% agarose, and imaged with CARS microscopy. Primary hepatocytes cultured on glass-bottom dishes were imaged directly with CARS microscopy without any preparation. On average, 9 liver volumetric analyses were performed per mouse and 9 mice per animal group were evaluated with CARS microscopy. At least 60 hepatocytes were evaluated for intracellular lipid content per experimental condition.

### Clinical blood lipid analysis

Analysis of blood lipid level (TAG, cholesterol, HDL, and LDL) were performed by Research Animal Diagnostic Laboratory (RADIL, Columbia, MO) on terminally collected blood samples of 9 mice per animal group. HDL and LDL were determined via direct measurement.

### Measurement of TAG with biochemical assays

Liver TAG levels for 9 mice per animal group were determined using a commercial Triglyceride Quantification Kit (Cat. No. 10010303, Cayman Chemical, Ann Arbor, MI) according to manufacturer's protocol and normalized with liver tissue weight.

### Measurement of FFA and TAG with LC-MS

Liver samples of approximately equal weight (∼40 mg) from each animal group were used for chloroform/methanol total lipid extraction. The recovered organic phases containing lipid were dried and reconstituted in equal volumes (100 µl) of chloroform/methanol. Approximately 50 µl of each liver lipid sample (3 mice per animal group) was used for evaluation of FFA and TAG with LC-MS as previously described [60].

### Measurement of NAD^+^/NADH and NADP^+^/NADPH ratios

NAD^+^/NADH and NADP^+^/NADPH ratios were measured from liver tissue lysates using commercially available kits according to manufacturer's protocols (Cat. No. K337-100 & K347-100, BioVision, Milpitas, CA). The liver tissues from at least 6 mice per animal group were used for measurement.

### Mitochondrial fraction isolation

Mitochondrial fraction was collected using a previously described protocol [61]. Liver tissues were immersed in 50 ml of ice cold isolation buffer and minced into small pieces. Isolation buffer (100 ml) was prepared with 10 ml of 0.1 M Tris-MOPS, 1 ml of EGTA/Tris, 20 ml of 1 M sucrose, and 69 ml of distilled water and adjusted pH to 7.4. Minced liver tissues were placed in a chilled Dounce homogenizer with fresh 5 ml of ice cold isolation buffer. Liver tissues were homogenized with 20–35 Dounce strokes. Homogenate was transferred to a 50 ml polypropylene Falcon tube and centrifuged at 600 g for 10 minutes at 4°C. Supernatant was transferred to a glass centrifuge tube and centrifuged at 7000 g for 10 minutes at 4°C. Supernatant was discarded and pellet washed with fresh 5 ml of ice cold isolation buffer. Glass tube was centrifuged again at 7000 g for 10 minutes at 4°C. Supernatant was discarded and pellet containing mitochondria fraction was re-suspended in 500 µl of isolation buffer supplemented with a protease inhibitor.

### Liver protein sample preparation

Frozen liver tissues at −80°C were crushed with mortar and pestle and placed in a tissue homogenizer on ice. Osmotic lysis buffer (10 mM Tris, pH 7.4, 0.3% SDS) containing protease inhibitor, nuclease, and phosphatase inhibitor was added and tissues were homogenized on ice. Freezed/thawed twice and placed on ice for 15 minutes. SDS Boiling Buffer (5% SDS, 5% BME, 10% glycerol and 60 mM Tris, pH 6.8) was added and placed in a water bath for 20 minutes. Tissues were cooled on ice, centrifuged to pellet solids, determined protein concentration, and stored at −80°C.

### 1D Western blots

Total liver protein extracts were separated on 10% SDS-PAGE gels, transferred to nitrocellulose membranes, incubated first with primary antibodies against proteins of interest and then with secondary antibodies conjugated with horseradish peroxidase (Cat. No. 31460, Thermo Scientific, Rockford, IL). Membranes were developed with enhanced chemiluminescence reagents (Cat. No. 34075, Thermo Scientific), stripped, and re-incubated with antibodies against β-actin or MnSOD for evaluation of loading controls. Primary antibodies were anti-acetylated lysine, anti-Sirt1, anti-Sirt3, and anti-β-actin from Cell Signaling (Cat. No. 9441, 2028, 5490, & 4967, Danvers, MA), MnSOD antibody from Millipore (Cat. No. 06-984, Billeria, MA), and anti-ECHD, anti-ACOX1, and anti-FABP1 from Abcam (Cat. No. ab72795, ab59964, ab7807, Cambridge, MA).

### 2D Western blots

2D Western blots were performed by Applied Biomics (Hayward, CA) and/or Kendrick Laboratories (Madison, WI). For 2D Western blots performed by Applied Biomics, 150 µg of protein from each liver tissue was loaded per gel and secondary antibodies were conjugated with Cy3 fluorescent dyes. For 2D Western blots performed by Kendrick Lab, 500 µg of protein from each liver tissue was loaded per gel and secondary antibodies were conjugated with horseradish peroxidase. Anti-acetylated lysine antibodies were provided by Applied Biomics and Kendrick Lab. Proteins were separated using isoelectric focusing (IEF) in the first dimension and SDS polyacrylamide gel electrophoresis (SDS-PAGE) in the second dimension. For 2D gels performed at Applied Biomics, proteins were labeled with CyDye DIGE fluors prior to 2D gel electrophoresis. At Kendrick Laboratory, isoelectric focusing was carried out in a glass tube of inner diameter 3.3 mm using 2.0% pH 4–8 mix Servalytes (Serva, Heidelberg, Germany; and 2 mM lysine) for 20,000 volt-hrs. After equilibration for 10 min in 10% glycerol, 50 mM dithiothreitol, 2.3% SDS and 0.0625 M tris, pH 6.8, each tube gel was sealed to the top of a stacking gel that overlaid a 10% acrylamide slab gel (1.00 mm thick). SDS slab gel electrophoresis was carried out for about 5 hrs at 25 mA/gel. The following proteins (Sigma Chemical Co., St. Louis, MO) were used as molecular weight standards: myosin (220,000), phosphorylase A (94,000), catalase (60,000), actin (43,000) carbonic anhydrase (29,000) and lysozyme (14,000). These standards appeared as bands at the basic edge of the Coomassie Brilliant Blue R-250-stained membrane.

### Protein identification with MALDI-TOF-MS

Immuno-positive protein spots were identified and corresponding protein spots from duplicate gels were picked and identified by MALDI-TOF-MS at Applied Biomics as described previously [5].

### Bioenergetics of primary hepatocytes

Immediately after isolation, primary hepatocytes were plated into 24-well plates at a density of 1×10^5^ cell per well. Plating media was consisted of DMEM with 25 mM glucose, 2 mM glutamine, 10% FBS, 0.1 mM sodium pyruvate, 1% Pen/Strep, and 1 mM HEPES at pH 7.4. At 4 hours after plating, primary hepatocytes were incubated for 24 hours with either uridine alone, drugs alone, or a combination of fenofibrate and uridine depending on the treatment condition. The final concentration used for uridine and/or fenofibrate was 100 µM. At 90 minutes prior to assaying, plating media was replaced with Cellular Assay Solution consisting of DMEM, 25 mM glucose, 2 mM glutamine, 1 mM sodium pyruvate and adjusted to pH 7.2 with 25 mM of MOPS. Bioenergetics of primary hepatocytes were determined using the XF Cell Mito Stress Test Kit and a XF24-3 Analyzer (Seahorse Bioscience, North Billerica, MA) following manufacturer's suggested protocols and published protocols [45]. Bioenergetics experiments were performed at the UCLA's Cellular Bioenergetics Core Facilities. At least 12 repeated measurements were performed per experimental condition. Final concentrations of oligomycin, FCCP, rotenone, and myxothiazol were 1 µg/ml, 1 µM, 0.1 µM, and 2 µM, respectively. Oxygen consumption rates were reported as absolute values (pmol O_2_ consumed per minute) on a per-unit of protein basis, where average protein concentration per well was normalized to 1.

### Statistical analysis

Data were presented as average values ± standard deviations. Statistical analysis was performed using Excel's paired Student t-test and analysis of variance (ANOVA) functions. Statistical significance was set at p≤0.05.

### Data availability

Selective MALDI-TOF-MS/MS spectra for protein identification, LC-MS spectra for free fatty acids quantification, and CARS imaging data are available via request to authors.

## Supporting Information

Figure S1 2D Western blots of acetylated proteins in liver total cell extracts of C57bl/6 mice. White arrows: ECHD; red arrows: FABP1. 2D Western blots were performed by Kendrick Laboratories.
(TIF)

Figure S2 2D Western blots of acetylated proteins in liver total cell extracts of *Sirt3*-KO mice. 2D Western blots were performed by Kendrick Laboratories.
(TIF)

Figure S3 Fatty acid β-oxidation measurement in primary hepatocytes. Rates of fatty acid β-oxidation was measured using a previously described protocol (Moon, A. & Rhead, J.W. ***J. Clin. Invest*** **.** **79**:59–64 (1987)). Briefly, [9,10(n)-^3^H] palmitic acid was added to plated primary hepatocyte cultures. Fatty acid β-oxidation was measured by monitoring the released ^3^H_2_O with a scintillation counter. The reaction rate was expressed as nmol ^3^H_2_O/mg protein/hour. The final concentrations of uridine and fenofibrate were 100 µM. The final concentration of tritiated palmitic acid and unlabeled palmitic acid mixture was 110 µM with specific radioactivity of 5-7E4 cpm/nmol. Error bars are standard deviation values across 6 repeated measurements per experimental condition.
(TIF)

Figure S4 Liver acetyl-CoA concentration as a function of fenofibrate and uridine treatment. Liver acetyl-CoA concentration is expressed as nmol per gram of liver weight. Liver acetyl-CoA were measured using commercial enzymatic assay kits according to manufacturer's protocols (Cat. No. ab87546, Abcam). Liver samples from at least six mice per animal group were used for evaluation. Triplicate measurements were performed per liver sample. *P<0.01 versus untreated control.
(TIF)

Figure S5 Immuno-fluorescence imaging of Sirt3 and peroxisomes. Sirt3 proteins were visualized via the use of primary antibodies against Sirt3 and secondary antibodies conjugated with FITC dye. Peroxisomes were visualized via the use of primary antibodies against catalase, a peroxisomal protein, and secondary antibodies conjugated to phycoerythrin (PE) dye. DAPI stains the DNA. (**A**) Images of a single frame along vertical axis. (**B**) 3D tiles of images taken along the vertical axis. Images were taken with 2-photon fluorescence microscopy using the CARS microscopy platform.
(TIF)

Table S1 Liver acetylated proteins identified with MALDI-TOF-MS.
(PDF)

Table S2 Liver acetylated proteins identified with MALDI-TOF-MS (continued 1).
(PDF)

Table S3 Liver acetylated proteins identified with MALDI-TOF-MS (continued 2).
(PDF)

Table S4 Protein acetylation sites identified with MALDI-TOF-MS-MS.
(PDF)

Table S5 Protein acetylation sites identified with MALDI-TOF-MS-MS (continued 1).
(PDF)

Table S6 Protein acetylation sites identified with MALDI-TOF-MS-MS (continued 2).
(PDF)
