# Supplementary figures and images for: Uridine Prevents Fenofibrate-Induced Fatty Liver
Source: PLoS One. 2014 Jan 24;9(1):e87179. doi: 10.1371/journal.pone.0087179 (PMC3901748; doi:10.1371/journal.pone.0087179)

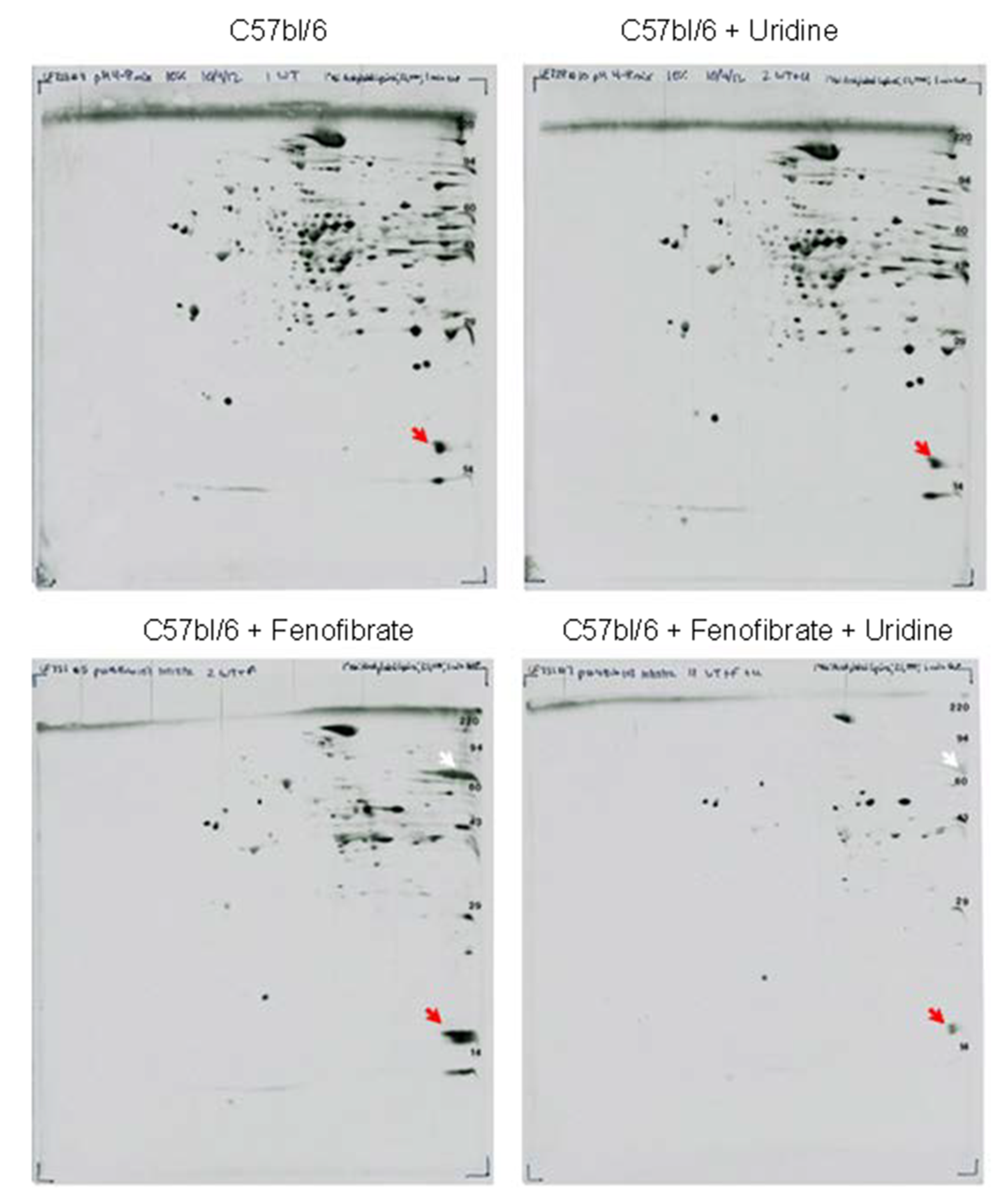

Supplement: Figure S1 — 2D Western blots of acetylated proteins in liver total cell extracts of C57bl/6 mice. White arrows: ECHD; red arrows: FABP1. 2D Western blots were performed by Kendrick Laboratories. (TIF) [file pone.0087179.s001.tif]

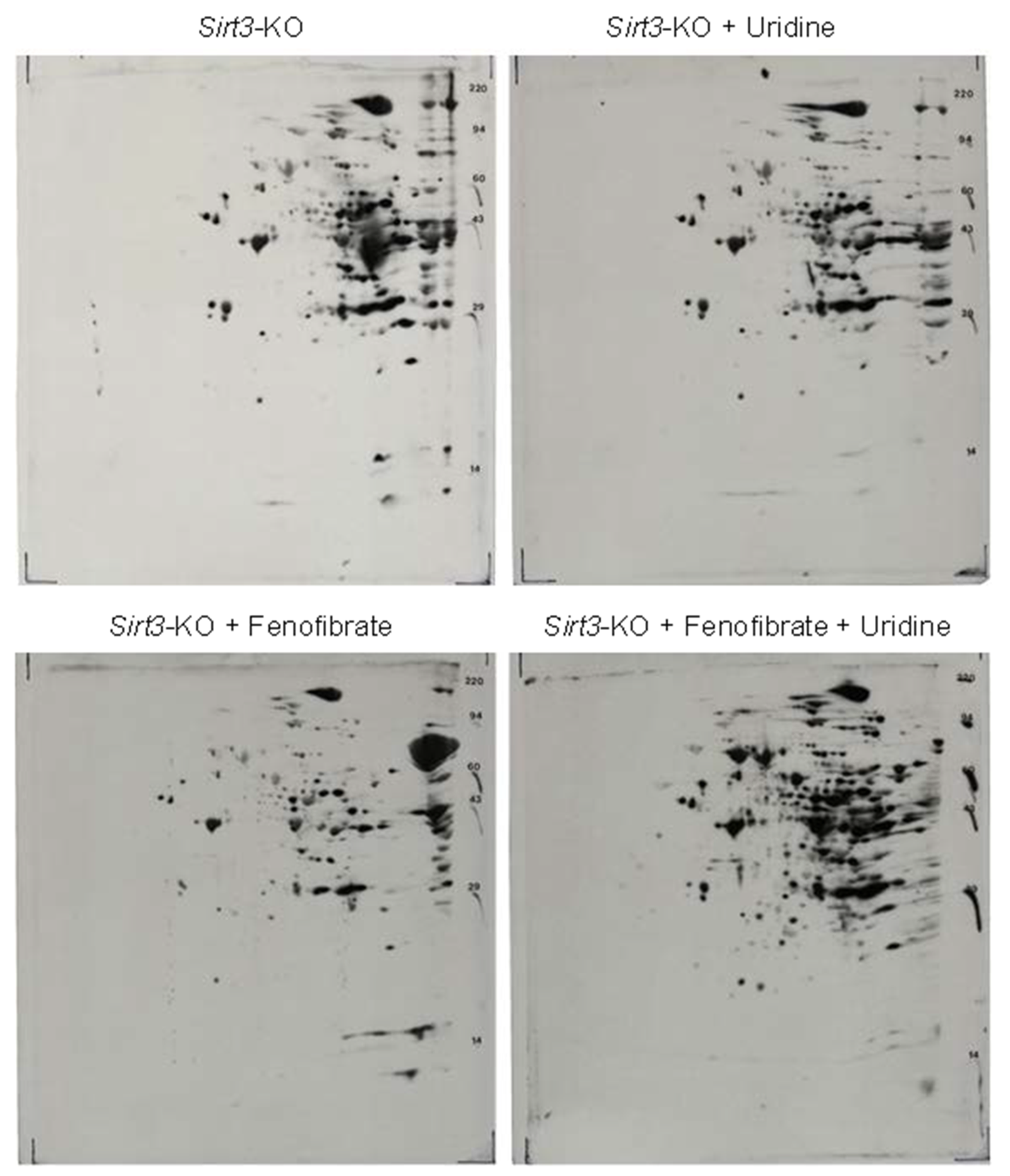

Supplement: Figure S2 — 2D Western blots of acetylated proteins in liver total cell extracts of Sirt3-KO mice. 2D Western blots were performed by Kendrick Laboratories. (TIF) [file pone.0087179.s002.tif]

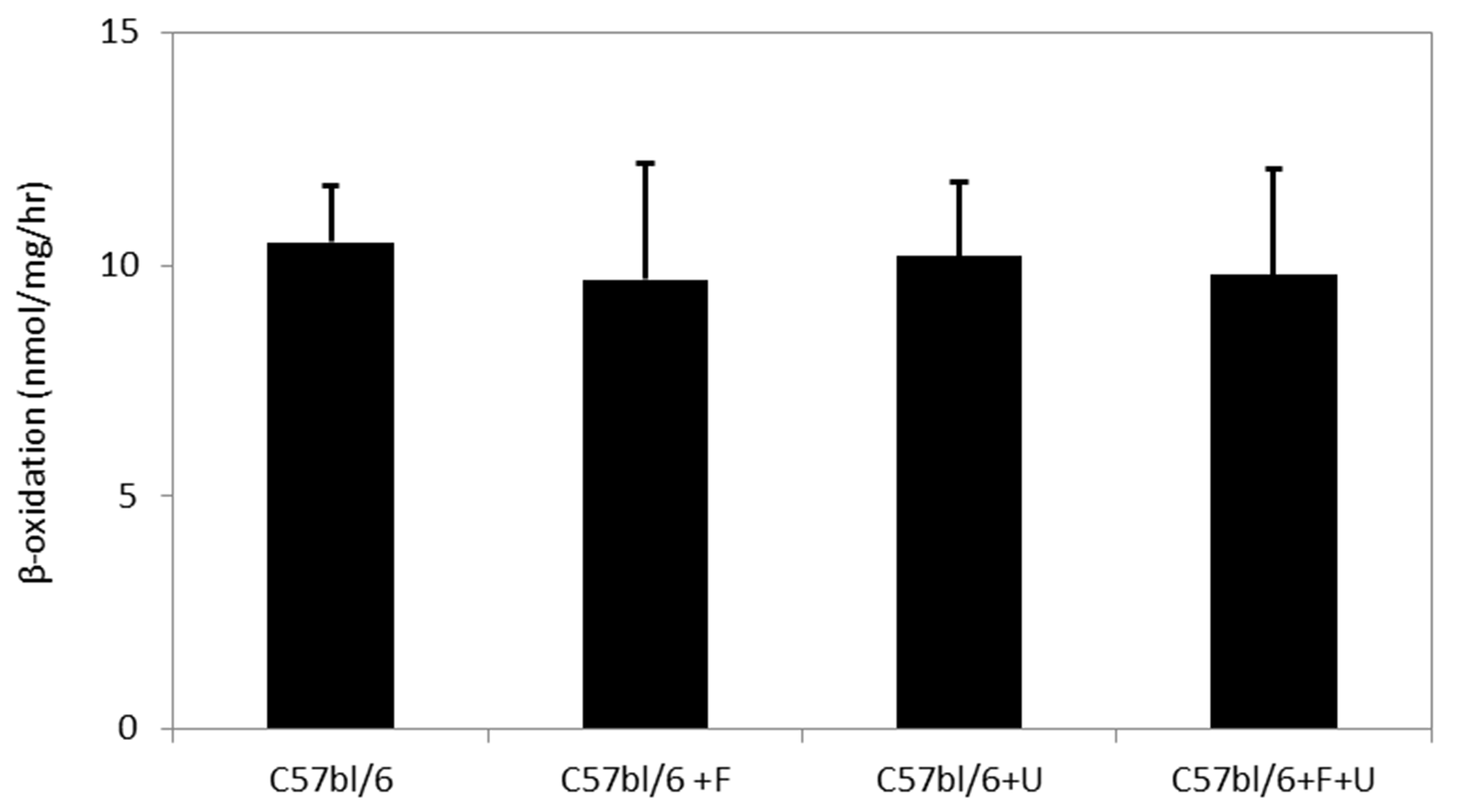

Supplement: Figure S3 — Fatty acid β-oxidation measurement in primary hepatocytes. Rates of fatty acid β-oxidation was measured using a previously described protocol (Moon, A. & Rhead, J.W. J. Clin. Invest . 79:59–64 (1987)). Briefly, [9,10(n)-3H] palmitic acid was added to plated primary hepatocyte cultures. Fatty acid β-oxidation was measured by monitoring the released 3H2O with a scintillation counter. The reaction rate was expressed as nmol 3H2O/mg protein/hour. The final concentrations of uridine and fenofibrate were 100 µM. The final concentration of tritiated palmitic acid and unlabeled palmitic acid mixture was 110 µM with specific radioactivity of 5-7E4 cpm/nmol. Error bars are standard deviation values across 6 repeated measurements per experimental condition. (TIF) [file pone.0087179.s003.tif]

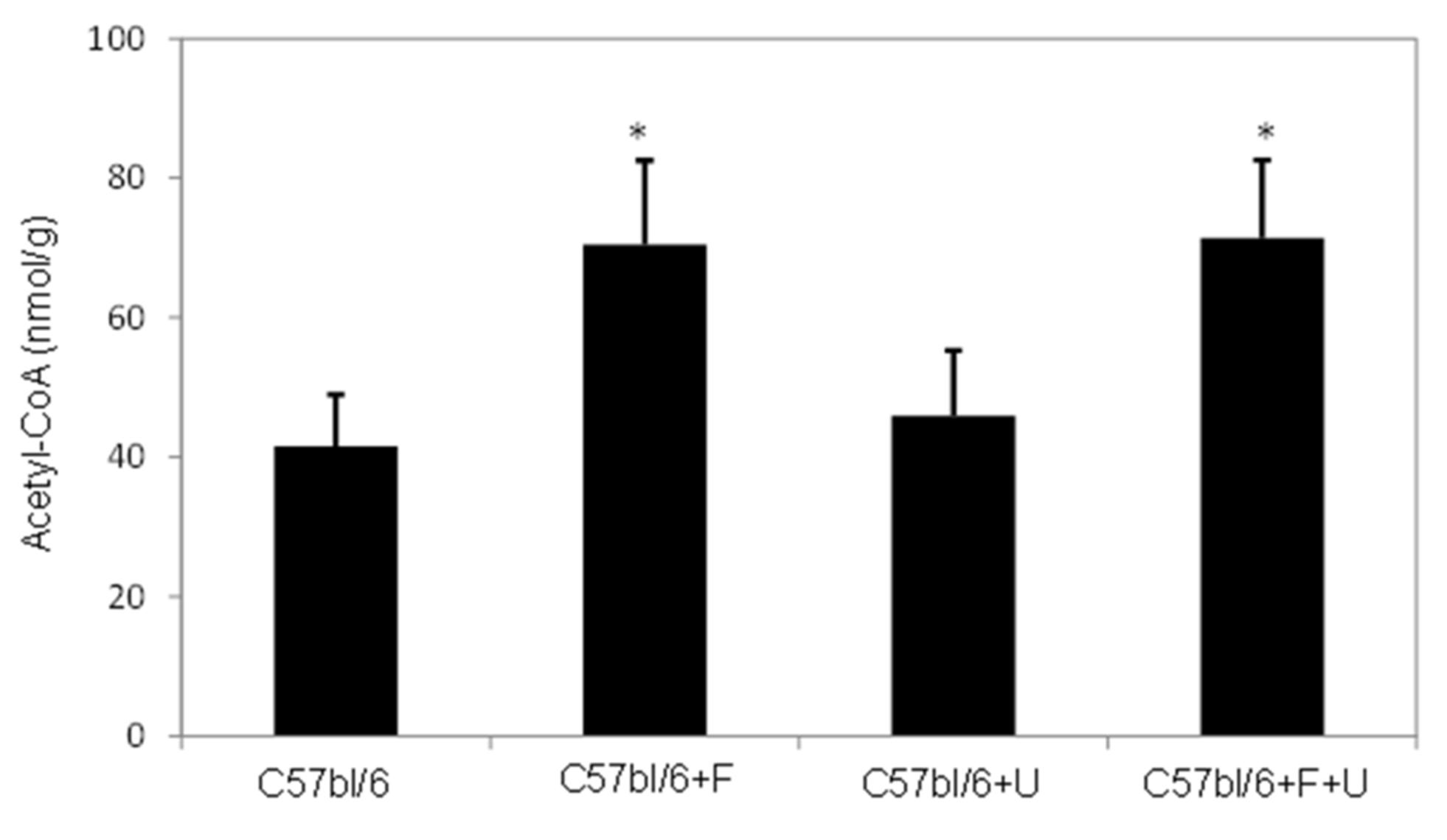

Supplement: Figure S4 — Liver acetyl-CoA concentration as a function of fenofibrate and uridine treatment. Liver acetyl-CoA concentration is expressed as nmol per gram of liver weight. Liver acetyl-CoA were measured using commercial enzymatic assay kits according to manufacturer's protocols (Cat. No. ab87546, Abcam). Liver samples from at least six mice per animal group were used for evaluation. Triplicate measurements were performed per liver sample. *P<0.01 versus untreated control. (TIF) [file pone.0087179.s004.tif]

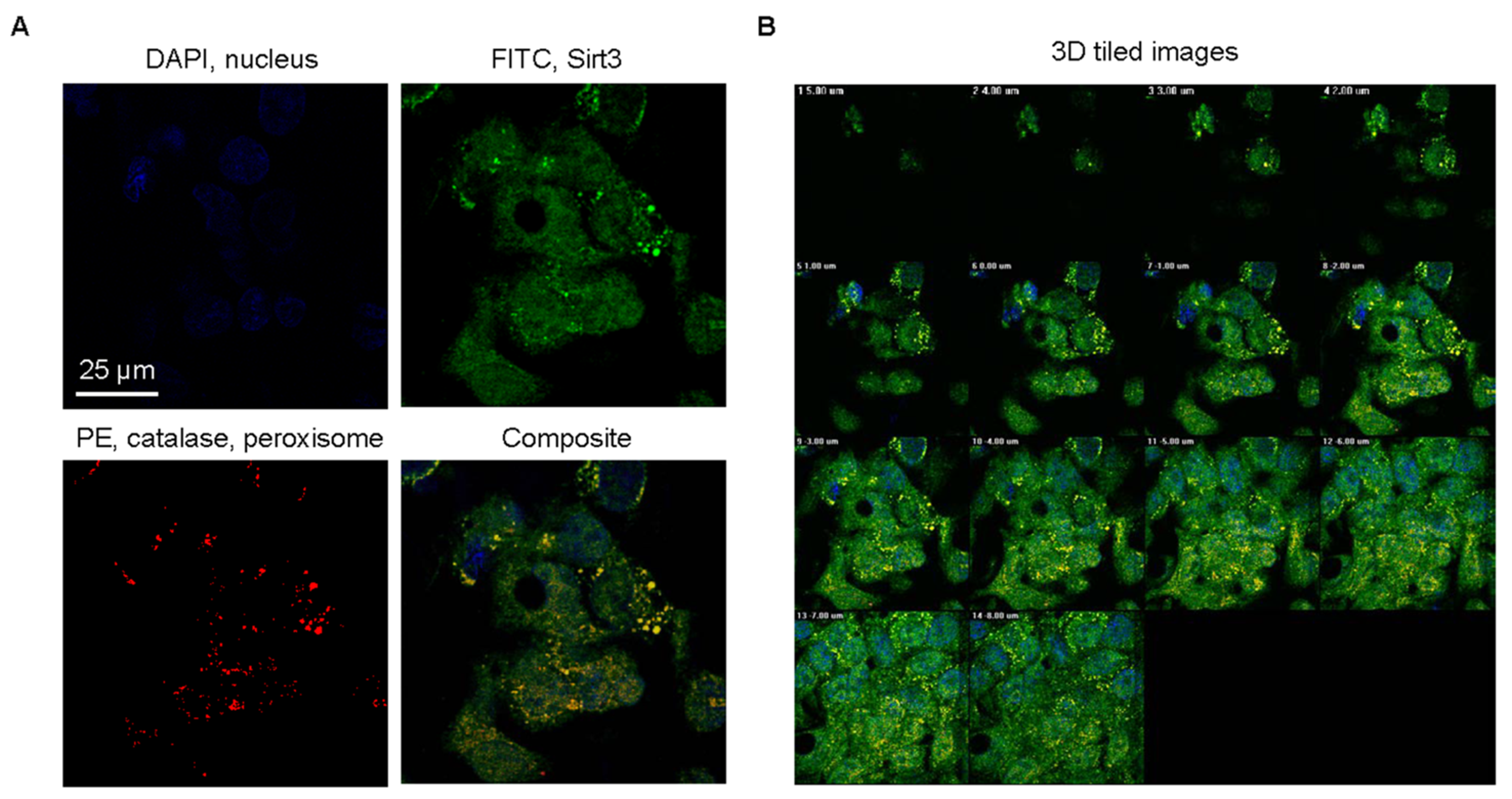

Supplement: Figure S5 — Immuno-fluorescence imaging of Sirt3 and peroxisomes. Sirt3 proteins were visualized via the use of primary antibodies against Sirt3 and secondary antibodies conjugated with FITC dye. Peroxisomes were visualized via the use of primary antibodies against catalase, a peroxisomal protein, and secondary antibodies conjugated to phycoerythrin (PE) dye. DAPI stains the DNA. (A) Images of a single frame along vertical axis. (B) 3D tiles of images taken along the vertical axis. Images were taken with 2-photon fluorescence microscopy using the CARS microscopy platform. (TIF) [file pone.0087179.s005.tif]
