# Supplementary material for: Uridine Prevents Fenofibrate-Induced Fatty Liver
Source: PLoS One. 2014 Jan 24;9(1):e87179. doi: 10.1371/journal.pone.0087179 (PMC3901748; doi:10.1371/journal.pone.0087179)
Supplement: Table S1 — Liver acetylated proteins identified with MALDI-TOF-MS. (PDF) [file pone.0087179.s006.pdf]

Table S1. Liver acetylated proteins identified with MALDI-TOF-MS

Spot #	Protein Name	Accession No.	Protein MW (Dalton)	Protein PI	Pep.Count	Protein Score	Protein Score C. I. %	Total Ion Score	Total Ion C. I. %
1	Endoplasmin	ENPL	92,418	4.7	34	1,120	100	856	100
2	78 kDa glucose-regulated protein	GRP78	72,378	5.1	29	1,230	100	1005	100
3	Cytosolic 10-formyltetrahydrofolate dehydrogenase	AL1L1	98,647	5.6	39	1,280	100	945	100
4	S-adenosylmethionine synthase isoform type-1	METK1	43,481	5.5	19	751	100	603	100
5	Aldehyde dehydrogenase, mitochondrial	ALDH2	56,502	7.5	19	750	100	623	100
6	Glutamate dehydrogenase 1, mitochondrial	DHE3	61,298	8.1	20	641	100	497	100
7	Glutamate dehydrogenase 1, mitochondrial	DHE3	61,298	8.1	21	688	100	535	100
8	Dihydropyrimidinase	DPYS	56,689	6.7	13	559	100	492	100
9	Catalase	CATA	59,758	7.7	28	1,110	100	856	100
10	Delta-1-pyrroline-5-carboxylate dehydrogenase	AL4A1	61,802	8.5	21	1,060	100	917	100
11	Methylmalonate-semialdehyde dehydrogenase	MMSA	57,879	8.3	21	911	100	754	100
12	SEC14-like protein 2	S14L2	46,271	6.7	11	121	100	72	100
13	Fumarylacetoacetase	FAAA	46,146	6.7	16	588	100	471	100
14	Isocitrate dehydrogenase [NADP] cytoplasmic	IDHC	46,645	6.7	20	608	100	473	100
15	Argininosuccinate synthase	ASSY	46,555	8.4	16	492	100	381	100
16	Betaine--homocysteine S-methyltransferase 1	BHMT1	44,992	8.0	17	454	100	336	100
17	Argininosuccinate synthase	ASSY	46,555	8.4	18	574	100	442	100
18	Argininosuccinate synthase	ASSY	46,555	8.4	17	638	100	515	100
19	Regucalcin	RGN	33,386	5.2	18	1,000	100	855	100
20	Indolethylamine N-methyltransferase	INMT	29,441	6.0	14	1,060	100	943	100
21	Glycine N-methyltransferase	GNMT	32,654	7.1	9	633	100	583	100
22	Carbonic anhydrase 3	CAH3	29,348	6.9	18	756	100	593	100
23	Carbonic anhydrase 3	CAH3	29,348	6.9	19	781	100	602	100
24	Superoxide dismutase [Mn], mitochondrial	SODM	24,588	8.8	7	149	100	108	100
25	Glutathione S-transferase	GSTM1	25,953	7.7	19	685	100	515	100
26	Histone H2B type 1-P	H2B1P	13,984	10.3	8	124	100	76	100
27	Catalase	CATA	59,758	7.7	26	1,090	100	870	100
28	Aldehyde dehydrogenase X, mitochondrial	AL1B1	57,517	6.6	3	53	92	39	99
29	Alpha-enolase	ENOA	47,111	6.4	15	629	100	534	100
30	Acyl-coenzyme A thioesterase 1	ACOT1	46,107	6.1	13	383	100	304	100
31	Arginase-1	ARGI1	34,786	6.5	18	643	100	492	100
32	Hydroxymethylglutaryl-CoA synthase, mitochondrial	HMCS2	56,787	8.7	12	190	100	138	100
33	Hydroxymethylglutaryl-CoA synthase, mitochondrial	HMCS2	56,787	8.7	19	799	100	683	100
34	Alcohol dehydrogenase 1	ADH1	39,746	8.4	12	413	100	347	100
35	3-ketoacyl-CoA thiolase B, peroxisomal	THIKB	43,968	8.8	20	1,100	100	934	100
