# Supplementary material for: Uridine Prevents Fenofibrate-Induced Fatty Liver
Source: PLoS One. 2014 Jan 24;9(1):e87179. doi: 10.1371/journal.pone.0087179 (PMC3901748; doi:10.1371/journal.pone.0087179)
Supplement: Table S2 — Liver acetylated proteins identified with MALDI-TOF-MS (continued 1). (PDF) [file pone.0087179.s007.pdf]

Table S2. **Liver acetylated proteins identified with MALDI-TOF-MS** (continued 1)

Spot #	Protein Name	Accession No.	Protein MW (Dalton)	Protein PI	Pep.Count	Protein Score	Protein Score C. I. %	Total Ion Score	Total Ion C. I. %
36	Malate dehydrogenase, mitochondrial	MDHM	35,589	8.9	11	456	100	390	100
37	Cytochrome c1, heme protein, mitochondrial	CY1	35,305	9.2	8	110	100	69	100
38	L-xylulose reductase	DCXR	25,729	6.8	10	281	100	215	100
39	D-beta-hydroxybutyrate dehydrogenase, mitochondrial	BDH	38,274	9.1	12	494	100	428	100
40	Heat shock cognate 71 kDa protein	HSP7C	70,827	5.4	27	1,090	100	874	100
41	ATP synthase subunit beta, mitochondrial	ATPB	56,266	5.2	25	1,130	100	900	100
42	Actin, cytoplasmic 2	ACTG	41,766	5.3	21	895	100	714	100
43	Fructose-bisphosphate aldolase B	ALDOB	39,482	8.5	18	953	100	807	100
44	Glyceraldehyde-3-phosphate dehydrogenase	G3PT	47,626	8.1	4	141	100	132	100
45	Uricase	URIC	35,017	8.5	16	665	100	546	100
46	Carbamoyl-phosphate synthase [ammonia], mitochondrial	CPSM	164,514	6.5	44	1,050	100	722	100
47	60 kDa heat shock protein, mitochondrial	CH60	60,917	5.9	27	1,380	100	1151	100
48	Epoxide hydrolase 2	HYES	62,475	5.9	27	1,160	100	932	100
49	NADP-dependent malic enzyme	MAOX	63,913	7.2	25	1,080	100	877	100
50	Peroxisomal acyl-coenzyme A oxidase 1	ACOX1	74,601	8.6	28	1,050	100	823	100
51	Peroxisomal bifunctional enzyme	ECHP	78,252	9.2	33	1,090	100	804	100
52	Peroxisomal bifunctional enzyme	ECHP	78,252	9.2	33	1,080	100	789	100
53	3-ketoacyl-CoA thiolase B, peroxisomal	THIKB	43,968	8.8	20	1,090	100	930	100
54	Elongation factor 1-alpha 1	EF1A1	50,082	9.1	16	463	100	358	100
55	Glycine N-acyltransferase-like protein	GLYAL	34,086	7.7	16	765	100	643	100
56	Hydroxyacyl-coenzyme A dehydrogenase, mitochondrial	HCDH	34,442	8.8	16	731	100	599	100
57	Electron transfer flavoprotein subunit beta	ETFB	27,606	8.2	10	610	100	554	100
58	Protein NipSnap homolog 1	NIPS1	33,342	9.5	16	726	100	602	100
59	Peroxisomal acyl-coenzyme A oxidase 1	ACOX1	74,601	8.6	13	745	100	690	100
60	Fatty acid-binding protein, liver	FABPL	14,237	8.6	8	462	100	400	100
61	Phosphoglucomutase-1	PGM1	61,380	6.1	19	402	100	283	100
62	4-trimethylaminobutyraldehyde dehydrogenase	AL9A1	53,480	6.6	19	544	100	424	100
63	Protein disulfide-isomerase A4	PDIA4	71,938	5.2	16	79	100	1	
64	Peroxisomal acyl-coenzyme A oxidase 1	ACOX1	74,601	8.6	30	1,070	100	816	100
65	Retinal dehydrogenase 1	AL1A1	54,433	7.9	26	798	100	583	100
66	Hydroxymethylglutaryl-CoA synthase, mitochondrial	HMCS2	56,787	8.7	20	755	100	627	100
67	Hydroxymethylglutaryl-CoA synthase, mitochondrial	HMCS2	56,787	8.7	14	65	99		
68	Alcohol dehydrogenase [NADP+]	AK1A1	36,564	6.9	19	727	100	560	100
69	Glycerol-3-phosphate dehydrogenase [NAD+]	GPDA	37,548	6.8	22	986	100	777	100
70	S-formylglutathione hydrolase	ESTD	31,299	6.7	5	87	100	67	100
