# Supplementary material for: Uridine Prevents Fenofibrate-Induced Fatty Liver
Source: PLoS One. 2014 Jan 24;9(1):e87179. doi: 10.1371/journal.pone.0087179 (PMC3901748; doi:10.1371/journal.pone.0087179)
Supplement: Table S3 — Liver acetylated proteins identified with MALDI-TOF-MS (continued 2). (PDF) [file pone.0087179.s008.pdf]

Table S3. **Liver acetylated proteins identified with MALDI-TOF-MS** (continued 2)

Spot #	Protein Name	Accession No.	Protein MW (Dalton)	Protein PI	Pep.Count	Protein Score	Protein Score C. I. %	Total Ion Score	Total Ion C. I. %
71	Superoxide dismutase [Mn], mitochondrial	SODM	24,588	8.8	13	482	100	373	100
72	Glutathione S-transferase	GSTM4	25,685	7.6	19	615	100	439	100
73	Glutathione S-transferase	GSTP1	23,594	7.7	8	457	100	401	100
74	Peptidyl-tRNA hydrolase 2, mitochondrial	PTH2	19,514	7.0	6	260	100	225	100
