# Supplementary material for: Uridine Prevents Fenofibrate-Induced Fatty Liver
Source: PLoS One. 2014 Jan 24;9(1):e87179. doi: 10.1371/journal.pone.0087179 (PMC3901748; doi:10.1371/journal.pone.0087179)
Supplement: Table S4 — Protein acetylation sites identified with MALDI-TOF-MS-MS. (PDF) [file pone.0087179.s009.pdf]

Table S4. Protein acetylation sites identified with MALDI-TOF-MS-MS

Sample Name	Observed Mass	Start seq.	End seq.	Acetylation-Peptide Sequence	Acetyl-Modification
**Peroxisomal acyl-coenzyme A oxidase 1**					
**Spot 50**	823.2278	250	255	ENMLMK	Acetyl (K)[6], Oxidation (M)[3]
**Accession No. ACOX1**	1758.6913	511	525	SKEVAWNLTSVDLVR	Acetyl (K)[2]
**M.W. 74,601; P.I. 8.6**	1769.6938	76	89	EFGIADPEEIMWFK	Acetyl (K)[14], Oxidation (M)[11]
	1949.8779	256	272	YAQVKPDGTYVKPLSNK	Acetyl (K)[5]
	2003.8507	526	542	ASEAHCHYVTVKVFADK	Acetyl (K)[12], Carbamidomethyl (C)[6]
	2218.9089	230	246	FGYEEMDNGYLKMDNYR	Acetyl (K)[12], Oxidation (M)[6,13]
	2780.2544	250	272	ENMLMKYAQVKPDGTYVKPLSNK	Acetyl (K)[6,11,18]
	2780.2544	250	272	ENMLMKYAQVKPDGTYVKPLSNK	Acetyl (K)[6,11,18]
	2780.7678	250	272	ENMLMKYAQVKPDGTYVKPLSNK	Acetyl (K)[6,11,18]
	2796.0886	250	272	ENMLMKYAQVKPDGTYVKPLSNK	Acetyl (K)[6,11,18], Oxidation (M)[3]
**Peroxisomal bifunctional enzyme**					
**Spot 51**	1368.5968	706	717	EWQSLAGPHSSK	Acetyl (K)[12]
**Accession No. ECHP**	1424.6086	573	583	GWYQYDKPLGR	Acetyl (K)[7]
**M.W. 78,252; P.I. 9.2**	1455.7605	241	252	HPYEVAIKEEAK	Acetyl (K)[8]
	1497.6047	182	194	FAQTVIGKPIEPR	Acetyl (K)[8]
	1585.7463	345	359	EASKSGQASAKPNLR	Acetyl (K)[4]
	2466.0591	173	194	SDPVEEAIKFAQTVIGKPIEPR	Acetyl (K)[9]
**Peroxisomal bifunctional enzyme**					
**Spot 52**	1341.6396	249	258	EEAKLFMYLR	Acetyl (K)[4]
**Accession No. ECHP**	1368.5953	706	717	EWQSLAGPHSSK	Acetyl (K)[12]
**M.W. 78,252; P.I. 9.2**	1424.6095	573	583	GWYQYDKPLGR	Acetyl (K)[7]
	1455.7596	241	252	HPYEVAIKEEAK	Acetyl (K)[8]
	1480.5819	34	46	NGLQKASLDHTVR	Acetyl (K)[5]
	1585.7349	345	359	EASKSGQASAKPNLR	Acetyl (K)[4]
	1991.9489	155	172	HISTDEALKLGILDVVVK	Acetyl (K)[9]
	2516.2593	654	676	HVGGPMYYAASVGLPTVLEKLQK	Acetyl (K)[20], Oxidation (M)[6]
	2568.2603	195	217	RILNKPVPSLPNMDSVFAEAIAK	Acetyl (K)[5], Oxidation (M)[13]
	2846.3704	360	384	FSSSTKELSSVDLVIEAVFEDMNLK	Acetyl (K)[6], Oxidation (M)[22]
