# Supplementary material for: Uridine Prevents Fenofibrate-Induced Fatty Liver
Source: PLoS One. 2014 Jan 24;9(1):e87179. doi: 10.1371/journal.pone.0087179 (PMC3901748; doi:10.1371/journal.pone.0087179)
Supplement: Table S5 — Protein acetylation sites identified with MALDI-TOF-MS-MS (continued 1). (PDF) [file pone.0087179.s010.pdf]

Supporting Information for

URIDINE PREVENTS FENOFIBRATE-INDUCED FATTY LIVER

Thuc T. Le[1, 2*], Yasuyo Urasaki[1, 2], Giuseppe Pizzorno[1, 2*]

[1]Nevada Cancer Institute, One Breakthrough Way, Las Vegas, NV 89135

[2]Desert Research Institute, 10530 Discovery Drive, Las Vegas, NV 89135, USA

[*]To whom correspondence should be addressed:

Thuc T Le, Desert Research Institute, 10530 Discovery Drive, Las Vegas, NV 89135, USA, Tel.: (702) 822-5223; Email: thuc@uchicago.edu

&

Giuseppe Pizzorno, Desert Research Institute, 10530 Discovery Way, Las Vegas, NV 89135, USA, Tel.: (702) 822-5380; Email: giuseppe.pizzorno@dri.edu

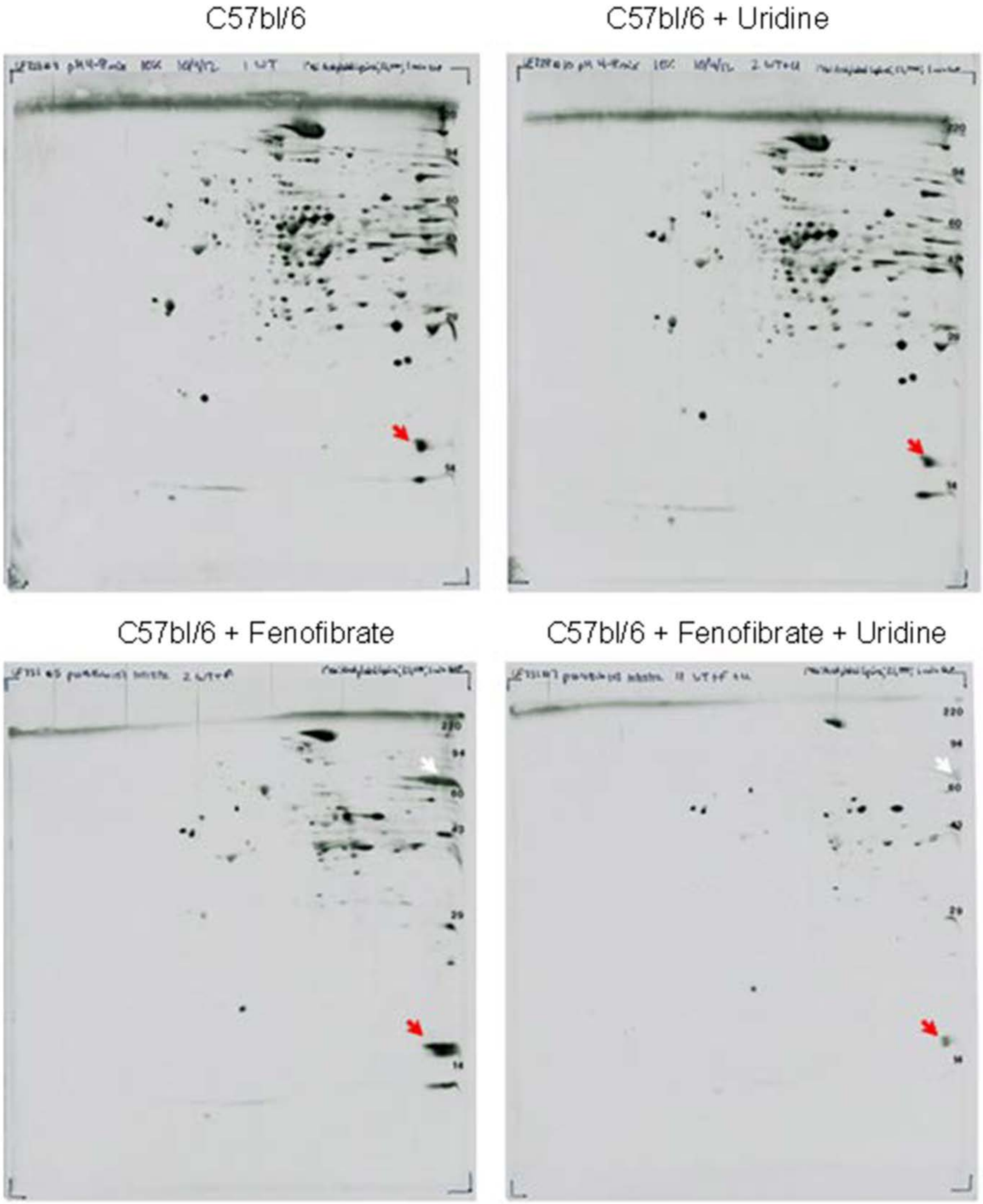

Figure S1. **2D Western blots of acetylated proteins in liver total cell extracts of C57bl/6 mice**. White arrows: ECHD; red arrows: FABP1. 2D Western blots were performed by Kendrick Laboratories.

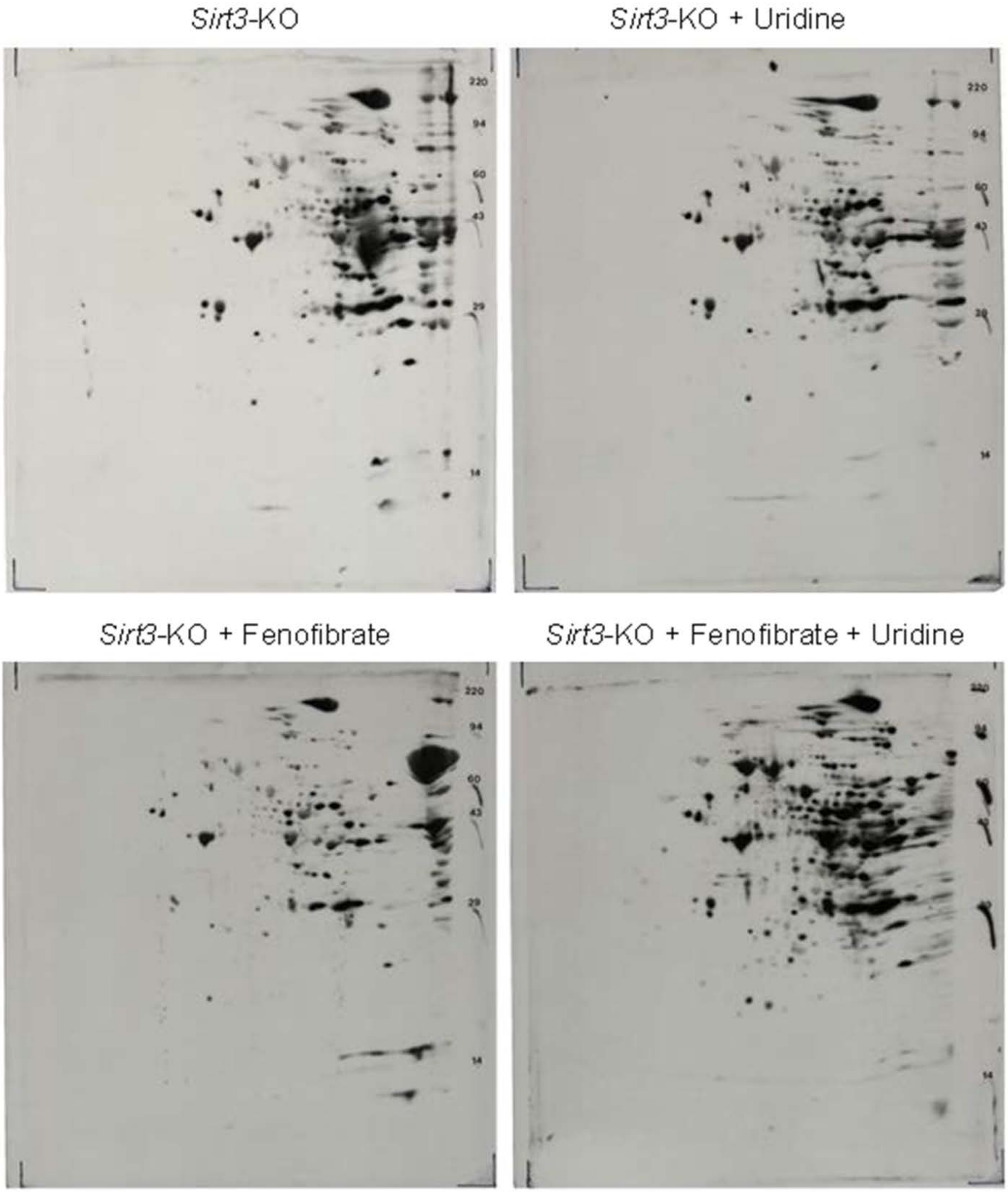

Figure S2. **2D Western blots of acetylated proteins in liver total cell extracts of *Sirt3*-KO mice**. 2D Western blots were performed by Kendrick Laboratories.

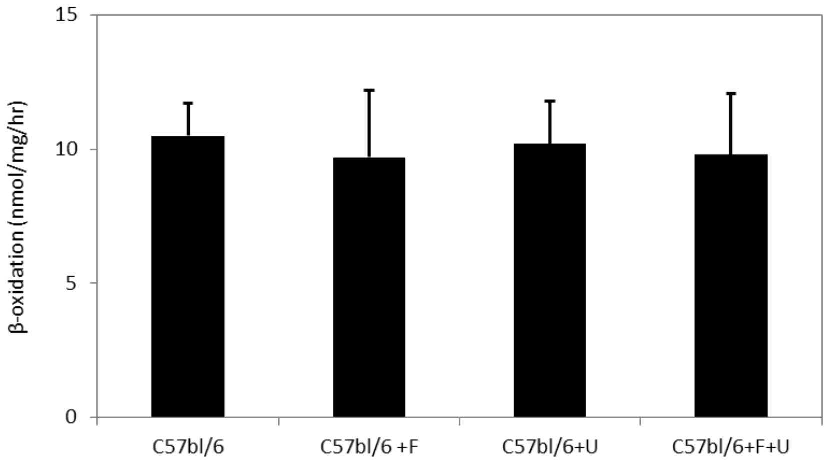

Figure S3. Fatty acid β-oxidation measurement in primary hepatocytes. Rates of fatty acid β-oxidation was measured using a previously described protocol (Moon, A. & Rhead, J.W. ***J. Clin. Invest.* 79**:59-64 (1987)). Briefly, [9,10(n)-^{3}H] palmitic acid was added to plated primary hepatocyte cultures. Fatty acid β-oxidation was measured by monitoring the released $^{3}H_2O$ with a scintillation counter. The reaction rate was expressed as nmol $^{3}H_2O$/mg protein/hour. The final concentrations of uridine and fenofibrate were 100 µM. The final concentration of tritiated palmitic acid and unlabeled palmitic acid mixture was 110 µM with specific radioactivity of 5-7E4 cpm/nmol. Error bars are standard deviation values across 6 repeated measurements per experimental condition.

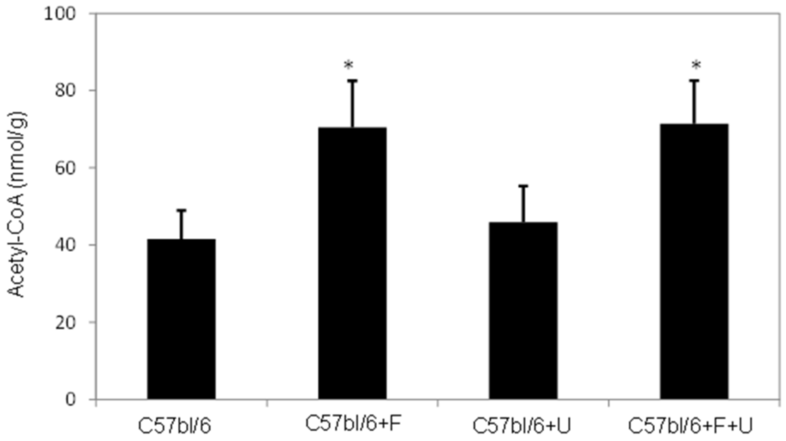

Figure S4. Liver acetyl-CoA concentration as a function of fenofibrate and uridine treatment. Liver acetyl-CoA concentration is expressed as nmol per gram of liver weight. Liver acetyl-CoA were measured using commercial enzymatic assay kits according to manufacturer's protocols (Cat. No. ab87546, Abcam). Liver samples from at least six mice per animal group were used for evaluation. Triplicate measurements were performed per liver sample. *P<0.01 versus untreated control.

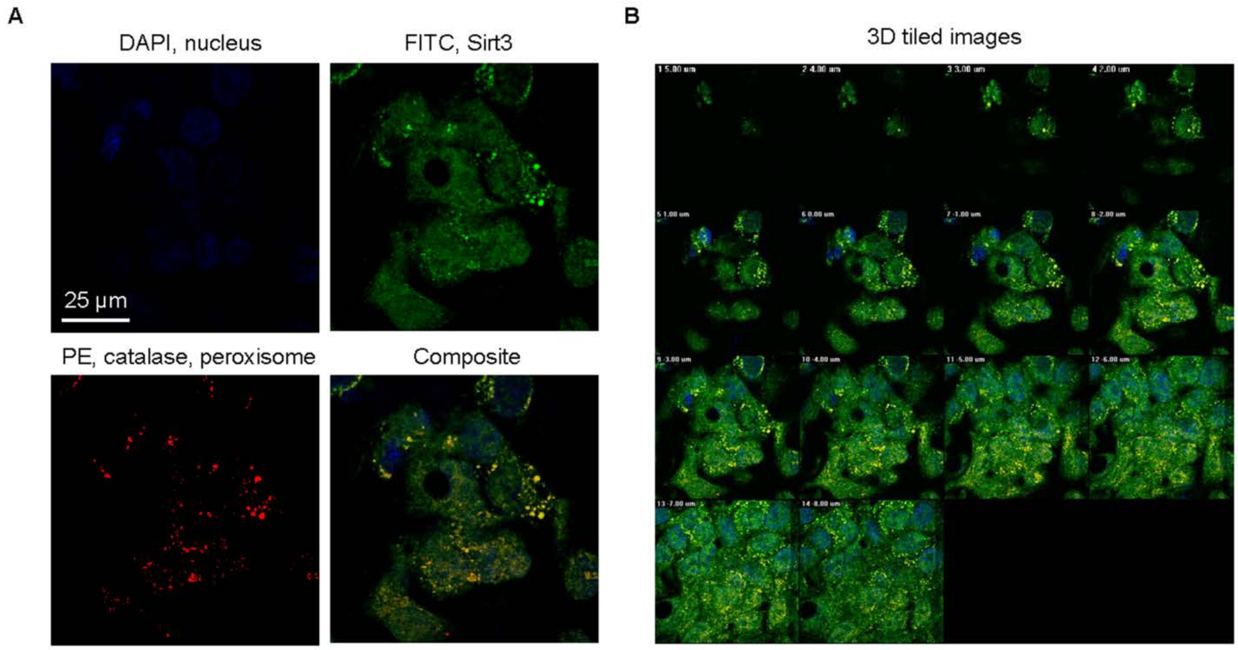

Figure S5. Immuno-fluorescence imaging of Sirt3 and peroxisomes. Sirt3 proteins were visualized via the use of primary antibodies against Sirt3 and secondary antibodies conjugated with FITC dye. Peroxisomes were visualized via the use of primary antibodies against catalase, a peroxisomal protein, and secondary antibodies conjugated to phycoerythrin (PE) dye. DAPI stains the DNA. (**A**) Images of a single frame along vertical axis. (**B**) 3D tiles of images taken along the vertical axis. Images were taken with 2-photon fluorescence microscopy using the CARS microscopy platform.

Table S1. Liver acetylated proteins identified with MALDI-TOF-MS

Spot #	Protein Name	Accession No.	Protein MW (Dalton)	Protein PI	Pep.Count	Protein Score	Protein Score C. I. %	Total Ion Score	Total Ion C. I. %
1	Endoplasmin	ENPL	92,418	4.7	34	1,120	100	856	100
2	78 kDa glucose-regulated protein	GRP78	72,378	5.1	29	1,230	100	1005	100
3	Cytosolic 10-formyltetrahydrofolate dehydrogenase	AL1L1	98,647	5.6	39	1,280	100	945	100
4	S-adenosylmethionine synthase isoform type-1	METK1	43,481	5.5	19	751	100	603	100
5	Aldehyde dehydrogenase, mitochondrial	ALDH2	56,502	7.5	19	750	100	623	100
6	Glutamate dehydrogenase 1, mitochondrial	DHE3	61,298	8.1	20	641	100	497	100
7	Glutamate dehydrogenase 1, mitochondrial	DHE3	61,298	8.1	21	688	100	535	100
8	Dihydropyrimidinase	DPYS	56,689	6.7	13	559	100	492	100
9	Catalase	CATA	59,758	7.7	28	1,110	100	856	100
10	Delta-1-pyrroline-5-carboxylate dehydrogenase	AL4A1	61,802	8.5	21	1,060	100	917	100
11	Methylmalonate-semialdehyde dehydrogenase	MMSA	57,879	8.3	21	911	100	754	100
12	SEC14-like protein 2	S14L2	46,271	6.7	11	121	100	72	100
13	Fumarylacetoacetase	FAAA	46,146	6.7	16	588	100	471	100
14	Isocitrate dehydrogenase [NADP] cytoplasmic	IDHC	46,645	6.7	20	608	100	473	100
15	Argininosuccinate synthase	ASSY	46,555	8.4	16	492	100	381	100
16	Betaine--homocysteine S-methyltransferase 1	BHMT1	44,992	8.0	17	454	100	336	100
17	Argininosuccinate synthase	ASSY	46,555	8.4	18	574	100	442	100
18	Argininosuccinate synthase	ASSY	46,555	8.4	17	638	100	515	100
19	Regucalcin	RGN	33,386	5.2	18	1,000	100	855	100
20	Indolethylamine N-methyltransferase	INMT	29,441	6.0	14	1,060	100	943	100
21	Glycine N-methyltransferase	GNMT	32,654	7.1	9	633	100	583	100
22	Carbonic anhydrase 3	CAH3	29,348	6.9	18	756	100	593	100
23	Carbonic anhydrase 3	CAH3	29,348	6.9	19	781	100	602	100
24	Superoxide dismutase [Mn], mitochondrial	SODM	24,588	8.8	7	149	100	108	100
25	Glutathione S-transferase	GSTM1	25,953	7.7	19	685	100	515	100
26	Histone H2B type 1-P	H2B1P	13,984	10.3	8	124	100	76	100
27	Catalase	CATA	59,758	7.7	26	1,090	100	870	100
28	Aldehyde dehydrogenase X, mitochondrial	AL1B1	57,517	6.6	3	53	92	39	99
29	Alpha-enolase	ENOA	47,111	6.4	15	629	100	534	100
30	Acyl-coenzyme A thioesterase 1	ACOT1	46,107	6.1	13	383	100	304	100
31	Arginase-1	ARGI1	34,786	6.5	18	643	100	492	100
32	Hydroxymethylglutaryl-CoA synthase, mitochondrial	HMCS2	56,787	8.7	12	190	100	138	100
33	Hydroxymethylglutaryl-CoA synthase, mitochondrial	HMCS2	56,787	8.7	19	799	100	683	100
34	Alcohol dehydrogenase 1	ADH1	39,746	8.4	12	413	100	347	100
35	3-ketoacyl-CoA thiolase B, peroxisomal	THIKB	43,968	8.8	20	1,100	100	934	100

Table S2. **Liver acetylated proteins identified with MALDI-TOF-MS** (continued 1)

Spot #	Protein Name	Accession No.	Protein MW (Dalton)	Protein PI	Pep.Count	Protein Score	Protein Score C. I. %	Total Ion Score	Total Ion C. I. %
36	Malate dehydrogenase, mitochondrial	MDHM	35,589	8.9	11	456	100	390	100
37	Cytochrome c1, heme protein, mitochondrial	CY1	35,305	9.2	8	110	100	69	100
38	L-xylulose reductase	DCXR	25,729	6.8	10	281	100	215	100
39	D-beta-hydroxybutyrate dehydrogenase, mitochondrial	BDH	38,274	9.1	12	494	100	428	100
40	Heat shock cognate 71 kDa protein	HSP7C	70,827	5.4	27	1,090	100	874	100
41	ATP synthase subunit beta, mitochondrial	ATPB	56,266	5.2	25	1,130	100	900	100
42	Actin, cytoplasmic 2	ACTG	41,766	5.3	21	895	100	714	100
43	Fructose-bisphosphate aldolase B	ALDOB	39,482	8.5	18	953	100	807	100
44	Glyceraldehyde-3-phosphate dehydrogenase	G3PT	47,626	8.1	4	141	100	132	100
45	Uricase	URIC	35,017	8.5	16	665	100	546	100
46	Carbamoyl-phosphate synthase [ammonia], mitochondrial	CPSM	164,514	6.5	44	1,050	100	722	100
47	60 kDa heat shock protein, mitochondrial	CH60	60,917	5.9	27	1,380	100	1151	100
48	Epoxide hydrolase 2	HYES	62,475	5.9	27	1,160	100	932	100
49	NADP-dependent malic enzyme	MAOX	63,913	7.2	25	1,080	100	877	100
50	Peroxisomal acyl-coenzyme A oxidase 1	ACOX1	74,601	8.6	28	1,050	100	823	100
51	Peroxisomal bifunctional enzyme	ECHP	78,252	9.2	33	1,090	100	804	100
52	Peroxisomal bifunctional enzyme	ECHP	78,252	9.2	33	1,080	100	789	100
53	3-ketoacyl-CoA thiolase B, peroxisomal	THIKB	43,968	8.8	20	1,090	100	930	100
54	Elongation factor 1-alpha 1	EF1A1	50,082	9.1	16	463	100	358	100
55	Glycine N-acyltransferase-like protein	GLYAL	34,086	7.7	16	765	100	643	100
56	Hydroxyacyl-coenzyme A dehydrogenase, mitochondrial	HCDH	34,442	8.8	16	731	100	599	100
57	Electron transfer flavoprotein subunit beta	ETFB	27,606	8.2	10	610	100	554	100
58	Protein NipSnap homolog 1	NIPS1	33,342	9.5	16	726	100	602	100
59	Peroxisomal acyl-coenzyme A oxidase 1	ACOX1	74,601	8.6	13	745	100	690	100
60	Fatty acid-binding protein, liver	FABPL	14,237	8.6	8	462	100	400	100
61	Phosphoglucomutase-1	PGM1	61,380	6.1	19	402	100	283	100
62	4-trimethylaminobutyraldehyde dehydrogenase	AL9A1	53,480	6.6	19	544	100	424	100
63	Protein disulfide-isomerase A4	PDIA4	71,938	5.2	16	79	100	1	
64	Peroxisomal acyl-coenzyme A oxidase 1	ACOX1	74,601	8.6	30	1,070	100	816	100
65	Retinal dehydrogenase 1	AL1A1	54,433	7.9	26	798	100	583	100
66	Hydroxymethylglutaryl-CoA synthase, mitochondrial	HMCS2	56,787	8.7	20	755	100	627	100
67	Hydroxymethylglutaryl-CoA synthase, mitochondrial	HMCS2	56,787	8.7	14	65	99		
68	Alcohol dehydrogenase [NADP+]	AK1A1	36,564	6.9	19	727	100	560	100
69	Glycerol-3-phosphate dehydrogenase [NAD+]	GPDA	37,548	6.8	22	986	100	777	100
70	S-formylglutathione hydrolase	ESTD	31,299	6.7	5	87	100	67	100

Table S3. **Liver acetylated proteins identified with MALDI-TOF-MS** (continued 2)

Spot #	Protein Name	Accession No.	Protein MW (Dalton)	Protein PI	Pep.Count	Protein Score	Protein Score C. I. %	Total Ion Score	Total Ion C. I. %
71	Superoxide dismutase [Mn], mitochondrial	SODM	24,588	8.8	13	482	100	373	100
72	Glutathione S-transferase	GSTM4	25,685	7.6	19	615	100	439	100
73	Glutathione S-transferase	GSTP1	23,594	7.7	8	457	100	401	100
74	Peptidyl-tRNA hydrolase 2, mitochondrial	PTH2	19,514	7.0	6	260	100	225	100

Table S4. Protein acetylation sites identified with MALDI-TOF-MS-MS

Sample Name	Observed Mass	Start seq.	End seq.	Acetylation-Peptide Sequence	Acetyl-Modification
**Peroxisomal acyl-coenzyme A oxidase 1**					
**Spot 50**	823.2278	250	255	ENMLMK	Acetyl (K)[6], Oxidation (M)[3]
**Accession No. ACOX1**	1758.6913	511	525	SKEVAWNLTSVDLVR	Acetyl (K)[2]
**M.W. 74,601; P.I. 8.6**	1769.6938	76	89	EFGIADPEEIMWFK	Acetyl (K)[14], Oxidation (M)[11]
	1949.8779	256	272	YAQVKPDGTYVKPLSNK	Acetyl (K)[5]
	2003.8507	526	542	ASEAHCHYVTVKVFADK	Acetyl (K)[12], Carbamidomethyl (C)[6]
	2218.9089	230	246	FGYEEMDNGYLKMDNYR	Acetyl (K)[12], Oxidation (M)[6,13]
	2780.2544	250	272	ENMLMKYAQVKPDGTYVKPLSNK	Acetyl (K)[6,11,18]
	2780.2544	250	272	ENMLMKYAQVKPDGTYVKPLSNK	Acetyl (K)[6,11,18]
	2780.7678	250	272	ENMLMKYAQVKPDGTYVKPLSNK	Acetyl (K)[6,11,18]
	2796.0886	250	272	ENMLMKYAQVKPDGTYVKPLSNK	Acetyl (K)[6,11,18], Oxidation (M)[3]
**Peroxisomal bifunctional enzyme**					
**Spot 51**	1368.5968	706	717	EWQSLAGPHSSK	Acetyl (K)[12]
**Accession No. ECHP**	1424.6086	573	583	GWYQYDKPLGR	Acetyl (K)[7]
**M.W. 78,252; P.I. 9.2**	1455.7605	241	252	HPYEVAIKEEAK	Acetyl (K)[8]
	1497.6047	182	194	FAQTVIGKPIEPR	Acetyl (K)[8]
	1585.7463	345	359	EASKSGQASAKPNLR	Acetyl (K)[4]
	2466.0591	173	194	SDPVEEAIKFAQTVIGKPIEPR	Acetyl (K)[9]
**Peroxisomal bifunctional enzyme**					
**Spot 52**	1341.6396	249	258	EEAKLFMYLR	Acetyl (K)[4]
**Accession No. ECHP**	1368.5953	706	717	EWQSLAGPHSSK	Acetyl (K)[12]
**M.W. 78,252; P.I. 9.2**	1424.6095	573	583	GWYQYDKPLGR	Acetyl (K)[7]
	1455.7596	241	252	HPYEVAIKEEAK	Acetyl (K)[8]
	1480.5819	34	46	NGLQKASLDHTVR	Acetyl (K)[5]
	1585.7349	345	359	EASKSGQASAKPNLR	Acetyl (K)[4]
	1991.9489	155	172	HISTDEALKLGILDVVVK	Acetyl (K)[9]
	2516.2593	654	676	HVGGPMYYAASVGLPTVLEKLQK	Acetyl (K)[20], Oxidation (M)[6]
	2568.2603	195	217	RILNKPVPSLPNMDSVFAEAIAK	Acetyl (K)[5], Oxidation (M)[13]
	2846.3704	360	384	FSSSTKELSSVDLVIEAVFEDMNLK	Acetyl (K)[6], Oxidation (M)[22]

Table S5. Protein acetylation sites identified with MALDI-TOF-MS-MS (continued 1)

Sample Name	Observed Mass	Start seq.	End seq.	Acetylation-Peptide Sequence	Acetyl-Modification
**Acyl-coenzyme A thioesterase**					
**Spot 30**	906.4299	321	327	NQVKMTK	Acetyl (K)[4], Oxidation (M)[5]
**Accession No. ACOT2**	1386.6136	370	380	SEFYADEISKR	Acetyl (K)[10]
**M.W. 49,626; P.I. 6.9**	1730.8285	258	274	SHPEVKGPGIGLLGISK	Acetyl (K)[6]
	2271.0911	325	344	MTKDGLLDVVEALQSPLVDK	Acetyl (K)[3,20], Oxidation (M)[1]
**Alcohol dehydrogenase Spot 34**	802.4053	325	331	SKDSVPK	Acetyl (K)[2]
**Accession No. ADH1**	976.4456	1	9	MSTAGKVIK	Acetyl (K)[6]
**M.W. 39,746; P.I. 8.4**	1053.5159	317	326	GAIFGGFKSK	Acetyl (K)[8]
	1295.7046	314	324	TWKGAIFGGFK	Acetyl (K)[3,11]
	1462.6885	103	114	ICKHPESNFCSR	Acetyl (K)[3]
	1576.6818	103	114	ICKHPESNFCSR	Acetyl (K)[3], Carbamidomethyl (C)[2,10]
	1576.6818	103	114	ICKHPESNFCSR	Acetyl (K)[3], Carbamidomethyl (C)[2,10]
	2001.9122	170	189	VCLIGCGFSTGYGSAVKVAK	Acetyl (K)[17]
	2600.2244	161	186	IDGASPLDKVCLIGCGFSTGYGSAVK	Acetyl (K)[9]
	3137.4448	12	38	AAVLWELHKPFTIEDIEVAPPKAHEVR	Acetyl (K)[9]
**3-ketoacyl-CoA thiolase B Spot 53**	2576.1960	78	102	LKPEQLGDISVGNVLQPGAGAIMAR	Acetyl (K)[2]
**3-ketoacyl-CoA thiolase B Spot 35**	2286.0649	217	237	GCFHAEIVPVTTTVLNDKGDK	Acetyl (K)[18]
**Accession No. THIKB**	2405.1692	238	259	KTITVSQDEGVRPSTTMQGLAK	Acetyl (K)[1], Oxidation (M)[17]
**M.W. 43,968; P.I. 8.8**					

Table S6. Protein acetylation sites identified with MALDI-TOF-MS-MS (continued 2)

Sample Name	Observed Mass	Start seq.	End seq.	Acetylation-Peptide Sequence	Acetyl-Modification
**Elongation factor 1α**					
**Spot 54**	803.3137	451	457	VTKSAQK	Acetyl (K)[3]
**Accession No. EF1A1**	828.4659	386	392	KLEDGPK	Acetyl (K)[1]
**M.W. 50,082; P.I. 9.1**	845.4504	451	457	VTKSAQK	Acetyl (K)[3,7]
	847.4164	31	37	CGGIDKR	Acetyl (K)[6], Carbamidomethyl (C)[1]
	847.4164	31	37	CGGIDKR	Acetyl (K)[6], Carbamidomethyl (C)[1]
	936.4746	38	44	TIEKFEK	Acetyl (K)[4]
	958.5259	314	321	NVSVKDVR	Acetyl (K)[5]
	958.5259	314	321	NVSVKDVR	Acetyl (K)[5]
	1051.6677	173	180	EVSTYIKK	Acetyl (K)[7,8]
	1496.6654	155	166	MDSTEPPYSQKR	Acetyl (K)[11], Oxidation (M)[1]
	1779.9117	70	84	GITIDISLWKFETSK	Acetyl (K)[10]
	1779.9117	70	84	GITIDISLWKFETSK	Acetyl (K)[10]
	1792.7578	21	36	STTTGHLIYKCGGIDK	Acetyl (K)[10], Carbamidomethyl (C)[11]
	2023.9440	248	266	LPLQDVYKIGGIGTVPVGR	Acetyl (K)[8]
	2557.2988	267	290	VETGVLKPGMVVTFAPVNVTTEVK	Acetyl (K)[7]
**Peroxisomal acyl-coenzyme A oxidase**					
**Spot 59**	1523.7189	230	241	FGYEEMDNGYLK	Acetyl (K)[12], Oxidation (M)[6]
**Accession No. ACOX1**	1614.7905	434	446	FLMKIYDQVQSGK	Acetyl (K)[4], Oxidation (M)[3]
**M.W. 74,601; P.I. 8.6**	1769.7092	76	89	EFGIADPEEIMWFK	Acetyl (K)[14], Oxidation (M)[11]
	1949.9733	256	272	YAQVKPDGTYVKPLSNK	Acetyl (K)[5]
	2460.2134	175	196	WWPGGLGKTSNHAIVLAQLITR	Acetyl (K)[8]
	2504.2402	273	295	LTYGTMVFVRSFLVGSAAQSLSK	Acetyl (K)[23]
	2780.3152	250	272	ENMLMKYAQVKPDGTYVKPLSNK	Acetyl (K)[6,11,18]
	2780.8413	250	272	ENMLMKYAQVKPDGTYVKPLSNK	Acetyl (K)[6,11,18]
**Fatty acid binding protein 1**					
**Spot 60**	1423.7704	21	33	AIGLPEDLIQKGK	Acetyl (K)[11]
**Accession No. FABPL**	2386.0815	61	80	NEFTLGEECELETMTGEKVK	Acetyl (K)[18], Carbamidomethyl (C)[9]
**M.W. 14,237; P.I. 8.6**	2402.0681	61	80	NEFTLGEECELETMTGE	Acetyl (K)[18], Carbamidomethyl (C)[9],
	2495.0247	1	20	MNFSGKYQLQSQENFEPFMK	Acetyl (K)[6]
	2495.0247	1	20	MNFSGKYQLQSQENFEPFMK	Acetyl (K)[6]
