# Supplementary material for: Uridine Prevents Fenofibrate-Induced Fatty Liver
Source: PLoS One. 2014 Jan 24;9(1):e87179. doi: 10.1371/journal.pone.0087179 (PMC3901748; doi:10.1371/journal.pone.0087179)
Supplement: Table S6 — Protein acetylation sites identified with MALDI-TOF-MS-MS (continued 2). (PDF) [file pone.0087179.s011.pdf]

Table S6. Protein acetylation sites identified with MALDI-TOF-MS-MS (continued 2)

Sample Name	Observed Mass	Start seq.	End seq.	Acetylation-Peptide Sequence	Acetyl-Modification
**Elongation factor 1α**					
**Spot 54**	803.3137	451	457	VTKSAQK	Acetyl (K)[3]
**Accession No. EF1A1**	828.4659	386	392	KLEDGPK	Acetyl (K)[1]
**M.W. 50,082; P.I. 9.1**	845.4504	451	457	VTKSAQK	Acetyl (K)[3,7]
	847.4164	31	37	CGGIDKR	Acetyl (K)[6], Carbamidomethyl (C)[1]
	847.4164	31	37	CGGIDKR	Acetyl (K)[6], Carbamidomethyl (C)[1]
	936.4746	38	44	TIEKFEK	Acetyl (K)[4]
	958.5259	314	321	NVSVKDVR	Acetyl (K)[5]
	958.5259	314	321	NVSVKDVR	Acetyl (K)[5]
	1051.6677	173	180	EVSTYIKK	Acetyl (K)[7,8]
	1496.6654	155	166	MDSTEPPYSQKR	Acetyl (K)[11], Oxidation (M)[1]
	1779.9117	70	84	GITIDISLWKFETSK	Acetyl (K)[10]
	1779.9117	70	84	GITIDISLWKFETSK	Acetyl (K)[10]
	1792.7578	21	36	STTTGHLIYKCGGIDK	Acetyl (K)[10], Carbamidomethyl (C)[11]
	2023.9440	248	266	LPLQDVYKIGGIGTVPVGR	Acetyl (K)[8]
	2557.2988	267	290	VETGVLKPGMVVTFAPVNVTTEVK	Acetyl (K)[7]
**Peroxisomal acyl-coenzyme A oxidase**					
**Spot 59**	1523.7189	230	241	FGYEEMDNGYLK	Acetyl (K)[12], Oxidation (M)[6]
**Accession No. ACOX1**	1614.7905	434	446	FLMKIYDQVQSGK	Acetyl (K)[4], Oxidation (M)[3]
**M.W. 74,601; P.I. 8.6**	1769.7092	76	89	EFGIADPEEIMWFK	Acetyl (K)[14], Oxidation (M)[11]
	1949.9733	256	272	YAQVKPDGTYVKPLSNK	Acetyl (K)[5]
	2460.2134	175	196	WWPGGLGKTSNHAIVLAQLITR	Acetyl (K)[8]
	2504.2402	273	295	LTYGTMVFVRSFLVGSAAQSLSK	Acetyl (K)[23]
	2780.3152	250	272	ENMLMKYAQVKPDGTYVKPLSNK	Acetyl (K)[6,11,18]
	2780.8413	250	272	ENMLMKYAQVKPDGTYVKPLSNK	Acetyl (K)[6,11,18]
**Fatty acid binding protein 1**					
**Spot 60**	1423.7704	21	33	AIGLPEDLIQKGK	Acetyl (K)[11]
**Accession No. FABPL**	2386.0815	61	80	NEFTLGEECELETMTGEKVK	Acetyl (K)[18], Carbamidomethyl (C)[9]
**M.W. 14,237; P.I. 8.6**	2402.0681	61	80	NEFTLGEECELETMTGE	Acetyl (K)[18], Carbamidomethyl (C)[9],
	2495.0247	1	20	MNFSGKYQLQSQENFEPFMK	Acetyl (K)[6]
	2495.0247	1	20	MNFSGKYQLQSQENFEPFMK	Acetyl (K)[6]
